# Characterization and Development of Microsatellite Markers in *Pseudotaxus chienii* (Taxaceae) Based on Transcriptome Sequencing

**DOI:** 10.3389/fgene.2020.574304

**Published:** 2020-10-15

**Authors:** Ruixiang Xu, Zhen Wang, Yingjuan Su, Ting Wang

**Affiliations:** ^1^School of Life Sciences, Sun Yat-sen University, Guangzhou, China; ^2^Research Institute of Sun Yat-sen University in Shenzhen, Shenzhen, China; ^3^College of Life Sciences, South China Agricultural University, Guangzhou, China

**Keywords:** *Pseudotaxus chienii*, transcriptome, microsatellite markers, genetic diversity, transferability

## Abstract

*Pseudotaxus chienii* (Taxaceae) is an endangered conifer species endemic to China. However, a lack of suitable molecular markers hinders the genomic and genetic studies on this species. Here, we characterized and developed the microsatellite markers from a newly sequenced *P. chienii* transcriptome. A total of 21,835 microsatellite loci were detected from 161,131 non-redundant unigene sequences, and the frequency of SSRs was 13.55%, with an average of one SSR loci per 9.18 kb. Mono-nucleotide, di-nucleotide, and tri-nucleotide were the dominant repeat types, accounting for 50.06, 13.49, and 29.39% of the total SSRs, respectively. In terms of distribution location, the coding regions (CDS) with few microsatellites and mainly consisted of tri-nucleotides. There were significant differences in the length of microsatellite among genic regions and motif types. Functional annotation showed that the unigenes containing microsatellites had a wide range of biological functions, most of which were related to basic metabolism, and a few might be involved in expression regulation of gene and response to environmental stress. In addition, 375 primer pairs were randomly selected and synthesized for the amplification and validation of microsatellite markers. Seventy-seven primer pairs were successfully amplified and 40 primer pairs were found to be polymorphic. Finally, 20 pairs of primers with high polymorphism were selected to assess the genetic diversity in four *P. chienii* populations. In addition, the newly developed microsatellite markers exhibited high transferability (70%) in *Amentotaxus argotaenia*. Our study could enable further genetic diversity analysis and functional gene mining on Taxaceae.

## Introduction

*Pseudotaxus chienii*, belonging to monotypic genus *Pseudotaxus* W. C. Cheng (Taxaceae) endemic to China, is narrowly distributed Zhejiang, Jiangxi, Hunan and Guangdong Province, and the Guangxi Zhuang Autonomous Region (Fu et al., 1999). The species is an evergreen shrub or small tree plants, up to 4 meters, characterized by seeds of a white fleshy aril (Fu et al., 1999). Most of its habitats are in the subtropical evergreen broad-leaved forest or deciduous broad-leaved forest at an elevation of about 800–1500 m (Fu et al., 1999). However, the distribution range and population size of *P. chienii* have been continuously declining due to the low seed-setting rate, the difficult natural reproduction, the strict habitat requirements, and the impact of human activities (Fu and Jin, 1992; Yang et al., 2005). Currently, *P. chienii* has been listed as a second-class nationally protected plant by the Chinese Red Data Book (Fu and Jin, 1992). Thus, it is imperative to develop effective strategies to protect this species. The first step is a comprehensive knowledge of its genetic diversity, which requires a powerful marker resource. However, due to the lack of genomic resources, the knowledge about functional gene analysis and molecular marker development of this species is limited.

Microsatellites, also known as simple sequence repeats (SSRs) or short tandem repeats (STRs), consist of short tandem repeated motifs of 1–6 bp units, which are ubiquitous in the genomes of prokaryotic and eukaryotic organisms (Tautz and Renz, 1984; Toth et al., 2000). Microsatellite markers are one of the most efficient types of molecular markers because of their high polymorphism, wide genome coverage, co-dominant inheritance, and good reproducibility (Powell et al., 1996; Gupta and Varshney, 2000; Varshney et al., 2005). Hence, they have been widely used in genetic linkage map, quantitative trait loci (QTL) mapping, marker-assisted selection (MAS) breeding, evolutionary studies, and genetic diversity analysis (Nishio et al., 2008; Garvin et al., 2010; Ahmadi and Fotokian, 2011; Wang et al., 2015; Zhao et al., 2019). Traditional methods for developing microsatellite markers are time-consuming, laborious, and expensive (Zane et al., 2002). With the development of next-generation sequencing technology (NGS), transcriptome sequencing has gradually become a convenient and efficient method for the development of large-scale SSR markers, especially for non-model species without an available reference genome (Taheri et al., 2018). SSRs can be divided into genomic SSRs (g-SSRs) and expressed sequence tag SSRs (EST-SSRs) (Eujayl et al., 2004). EST-SSRs are derived from gene transcription regions and are more conserved than g-SSRs, therefore, are more likely to be transferable across related species (Dutta et al., 2011). To date, there have been few investigations of EST-SSR markers in *P. chienii*, especially based on transcriptome sequencing.

The distribution of microsatellites in genomic regions is non-random (Li et al., 2002). For example, in many species, protein-coding regions contain fewer di-nucleotide and tetra-nucleotide SSRs, but more tri-nucleotide and hexa-nucleotide SSRs (Li et al., 2004). According to a study on SSRs in human and *Arabidopsis thaliana*, the number of SSRs in untranslated regions (UTRs) was more abundant than that in coding regions (CDS) (Zhang et al., 2004). In general, the occurrence frequency of SSRs in coding regions is relatively low, which may be explained by its high mutation rate affecting gene expression (Vieira et al., 2016). Besides, similar distribution patterns were observed in microsatellites located in intergenic regions and introns (Hancock, 1995).

The function of microsatellites is associated with their location in the genome. Compared with other genic regions, microsatellites in coding regions are the most conserved (Xin et al., 2012), which may be attributed to selection pressure (Li et al., 2004). Microsatellite variations in coding regions can directly affect the protein synthesis process (Gao et al., 2013). SSR variations in 5′-UTRs can be involved in gene expression regulation by affecting transcription and translation (Li et al., 2004; Vieira et al., 2016). SSR expansions in 3′-UTRs can lead to transcription slippage and mRNA sequence expansion, which further disrupt mRNA splicing and other cell functions (Li et al., 2004; Kalia et al., 2011). Intronic SSRs may play a role in regulating gene transcription, mRNA splicing, or export to cytoplasm (Li et al., 2004). SSR variations in intergenic regions may have an impact on gene function by changing the secondary structure of DNA or the source of small RNAs (Gao et al., 2013). However, there has been no report on the function of microsatellites in *P. chienii*, which is not conducive to the discovery of functional genes and the identification of phenotypic variation.

In this study, we mined microsatellite loci from a newly sequenced transcriptome of *P. chienii* using Illumina sequencing technology. The aims of this study were to (i) characterize the frequency, distribution, function, and evolution of microsatellites in *P. chienii* transcriptome; (ii) develop polymorphic microsatellite markers and verified their polymorphism level for *P. chienii*; (iii) test cross-species transferability of the novel microsatellite markers among Taxaceae species. The results of this study will provide new insights into the function and evolution of microsatellites in *P. chienii* transcriptome. Additionally, the newly developed microsatellite markers may facilitate the conservation and management of *P. chienii* genetic resources.

## Materials and Methods

### Sample Collection, RNA and DNA Extraction

The plant materials (root, stem, leaf, and strobilus) used for RNA isolation and transcriptome sequencing were collected from a single *Pseudotaxus chienii* individual growing in Bijiashan, Jiangxi Province, China (26°30′37′′N, 114°09′42′′E, 1290 m asl). Each tissue sample was immediately stored in RNA Protective Additive after collection and removal of dust. The collected samples were first stored overnight at 4°C and then stored at −20°C the next day. Total RNA was separately extracted from each tissue using the RNAprep pure Plant Kit (Tiangen, Beijing, China) according to the manufacturer’s instructions. RNA degradation and contamination were detected using 1% agarose gels. RNA quality assessments for purity, integrity, and concentration were separately performed using the NanoPhotometer spectrophotometer (IMPLEN, CA, United States), Qubit 2.0 Fluorometer (Life Technologies, CA, United States), and Agilent Bioanalyzer 2100 system (Agilent Technologies, CA, United States).

For SSR characterization, the young leaves of 110 individuals were collected from four geographic localities across China: 31 from Maoshan, Zhejiang (MS), 30 from Yinshan park, Guangxi (YS), 30 from Bijiashan, Jiangxi (BJS), and 19 from Zhangjiajie, Hunan (ZJJ) (Table 1). Total genomic DNA was extracted using the modified cetyltrimethyl ammonium bromide (CTAB) method (Su et al., 2005). The quality and quantity of DNA were determined by NanoDrop 2000 spectrophotometer (ThermoFisher Scientific, Waltham, MA, United States) and 0.8% agarose gel electrophoresis. Finally, all DNA samples were diluted to 50 ng/μL and stored at −20°C until use.

**TABLE 1:** Sampling location information of four *P. chienii* populations.

Population	Location	Sample Size	Latitude (N)	Longitude (E)	Altitude (m a.s.l.)
MS	Maoshan, Zhejiang	31	118°58′23′′	28°06′08′′	1120
YS	Yinshan park, Guangxi	30	110°14′53′′	24°09′15′′	1050
BJS	Bijiashan, Jiangxi	30	114°09′41′′	26°30′35′′	1340
ZJJ	Zhangjiajie, Hunan	19	110°28′56′′	29°23′12′′	1055

### Transcriptome Sequencing and *de novo* Assembly

The qualified RNA samples from four tissues were used for cDNA library construction and transcriptome sequencing. Four cDNA libraries were constructed using NEBNext Ultra RNA Library Prep Kit for Illumina (NEB, United States) according to the manufacturer’s instructions. Transcriptome sequencing was performed using Illumina HiSeq 2500 platform (Illumina, San Diego, CA, United States). After sequencing, raw reads were filtered to remove the adaptor sequences and low-quality reads. The remaining high-quality clean reads were *de novo* assembled using Trinity software (Grabherr et al., 2011) with min_kmer_cov set to 2 and other parameters set to default. Assembled transcripts were clustered, and removal of the redundant sequences to generate non-redundant unigenes by CD-HIT software (Fu et al., 2012).

### Microsatellite Identification and Characterization

The MISA software (Microsatellite searching tool^1^) was used to identify microsatellite loci in the assembled transcriptome of *P. chienii*. The parameters were set as follows: mono-, di-, tri-, tetra-, penta-, and hexa-nucleotides with minimum repeat numbers of 10, 6, 5, 5, 5, and 5, respectively. For compound SSRs, the maximum interruption between two SSRs was set as 100 bases.

In order to investigate the distribution of microsatellites in *P. chienii* transcriptome, the coding sequence (CDS) regions of unigenes were predicted based on the BLAST results against the Swiss-Prot database (*E*-value < 10^–5^), and then CDS regions that showed no hits in BLAST were predicted using ESTScan (Iseli et al., 1999). The relative position of microsatellites (CDS, 5′-UTR, or 3′-UTR) was inferred from the positions of start and stop codons of the CDS. Chi-square analysis was used to test whether there were significant differences in different genic regions (CDS, 5′-UTR, and 3′-UTR) for the density of microsatellites. Kruskal–Wallis rank sum test (Kruskal and Wallis, 1952) was used to test the influence of genic region, repeat motif size, and repeat motif type on microsatellite length.

### Function Annotation of Unigenes Containing Microsatellites

To identify the possible function of microsatellites, all unigene sequences containing microsatellites were aligned to the public databases by BLASTX program (*E*-value < 10^–5^), including Nr (NCBI non-redundant protein sequences), Nt (NCBI non-redundant nucleotide sequences), Swiss-Prot (A manually annotated and reviewed protein sequence database), KOG (Clusters of eukaryotic Orthologous Groups), and Pfam (Protein family, assigned using the HMMER3.0 package). Besides, for all unigene sequences containing microsatellites, the GO (Gene Ontology) annotation was performed by the Blast2GO software (Conesa et al., 2005) (*E*-value < 10^–6^). The metabolic pathway analysis was predicted by KAAS (KEGG Automatic Annotation Server) (*E*-value < 10^–10^).

To determine whether microsatellites were significantly enriched in some GO terms or KEGG pathways, GO and KEGG enrichment analysis were performed with the topGO package (Alexa and Rahnenfuhrer, 2010) and clusterProfiler package (Yu et al., 2012), respectively. Over-represented GO terms/KEGG pathways were estimated by Fisher’s exact test with multiple testing correction of FDR (*Q*-value < 0.05) (Benjamini and Hochberg, 1995).

### Amplification and Validation of Microsatellite Markers

Microsatellite primers were designed using Primer Premier 5.0 (Lalitha, 2000) based on the following criteria: (1) primer length of 18–25 bp, with an optimum length of 20 bp; (2) annealing temperature of 50–62°C with a maximum of 5°C difference in annealing temperature between forward and reverse primers; (3) CG content from 40 to 60%; (4) the expected PCR product sizes of 100–500 bp; (5) avoidance of primer dimers and hairpin structures.

PCR amplifications were conducted in a volume of 25 μL containing 50 ng of template DNA, 2.5 μL of 10 × PCR buffer (2.0 mM Mg^2+^), 1.6 μL of dNTPs (10 mM), 0.5 μL of each primer (10 μM), 1 U of Taq polymerase, and 18.7 μL of double-distilled water. The PCR conditions were as follows: initial denaturation at 94°C for 5 min, followed by 35 cycles of 40 s at 94°C, 40 s at annealing temperature, and 30 s at 72°C, and a final extension at 72°C for 10 min. The amplified products were detected by 1.5% (w/v) agarose gel electrophoresis. Then, the successfully amplified primers were selected for polymorphic screening using 6% denaturing polyacrylamide gel electrophoresis. Finally, microsatellite primers with high polymorphism and good repeatability were selected for genotyping on an ABI 3730XL DNA Analyzer (Applied Biosystems, Foster City, CA, United States) with GeneScan LIZ 500 as an internal reference (Applied Biosystems). Allele sizes were detected with the software GeneMapper v4.0 (Applied Biosystems).

The genetic parameters of microsatellite loci were estimated by GenAlEx v6.5 software (Peakall and Smouse, 2012), including the number of alleles (Na), the number of effective alleles (Ne), observed heterozygosity (Ho), and expected heterozygosity (He). The polymorphic information content (PIC) of each microsatellite marker was calculated using PIC_CALC v0.6 (Nagy et al., 2012). The null allele frequency for each marker was estimated using MICRO-CHECKER 2.2.3 (Van Oosterhout et al., 2004). Tests for deviations from linkage disequilibrium (LD) and Hardy-Weinberg equilibrium (HWE) were carried out by GENEPOP v4.2 (Raymond and Rousset, 1995). The software BOTTLENECK v1.2.02 (Piry et al., 1999) was used to detect recent population bottlenecks under three different models of microsatellite evolution (Infinite allele model, IAM; Stepwise mutation model, SMM; Two-phased model of mutation, TPM).

### Cross-Species Amplification and Transferability Analysis

For cross-species transferability analysis, the related species of the family Taxaceae, *Amentotaxus argotaenia* (with 58 individuals from four different populations, Supplementary Table 1), was chosen to evaluate the transferability of these newly developed microsatellite markers. Genomic DNA isolation and PCR amplification were performed as described above. PCR products were separated by capillary electrophoresis using an ABI 3730XL DNA Analyzer (Applied Biosystems, Foster City, CA, United States), and the sizes of fragments were determined using GeneMapper v4.0 (Applied Biosystems).

## Results

### Illumina Sequencing and *de novo* Assembly

In this study, the Illumina HiSeq 2500 platform was used to sequence the transcriptome of *P. chienii*. A total of 52,633,598, 57,112,536, 62,840,720, and 42,617,842 raw reads were generated from root, stem, leaf, and strobilus, respectively. After trimming adaptors and removing low-quality reads, 51,073,864, 56,419,624, 61,110,258, 41,448,574 high-quality clean reads were obtained for root, stem, leaf, and strobilus (Table 2). The total nucleotide number of each tissue was greater than 6G. The Q20 and Q30 ratio of each tissue were over 95 and 91%, respectively. The GC content of each tissue was approximately 45% (Table 2). Thus, the sequencing data was of high quality and met the requirement for subsequent analyses. Using the Trinity program (Grabherr et al., 2011), the high-quality clean reads from four tissue were assembled into 156,747, 202,908, 107,675, and 201,124 transcripts, respectively. The assembled transcripts of these tissues were clustered into 66,126, 79,842, 52,207, and 60,391 unigenes (Table 2). After removing the redundant sequences, a total of 161,131 non-redundant unigenes were generated.

**TABLE 2:** Summary of *de novo* transcriptome assembly of four tissues from *P. chienii*.

Categories	Items	Root	Stem	Leaf	Strobilus
Raw reads	Total raw reads	52,633,598	57,112,536	62,840,720	42,617,842
Clean reads	Total clean reads	51,073,864	56,419,624	61,110,258	41,448,574
	Total clean bases (Gb)	7.66	8.46	9.17	6.22
	Q20 (%)	97.16	98.03	96.9	97.28
	Q30 (%)	92.16	94.03	91.55	92.34
	GC content (%)	44.25	46.26	45.57	45.23
Transcripts	Total number of transcripts	156,747	202,908	107,675	201,124
	Total length of transcripts (bp)	113,815,935	136,683,283	89,744,115	114,523,821
	Maximum length of transcripts (bp)	13,579	12,558	11,562	12,275
	Minimum length of transcripts (bp)	201	201	201	201
	Mean length of transcripts (bp)	726	674	833	569
	N50 (bp)	1,395	1,224	1,621	1,011
Unigenes	Total number of unigenes	66,126	79,842	52,207	60,391
	Total length of unigenes (bp)	87,790,725	101,369,156	74,169,328	76,205,289
	Maximum length of unigenes (bp)	13,579	12,558	11,562	12,275
	Minimum length of unigenes (bp)	201	201	201	201
	Mean length of unigenes (bp)	1,328	1,270	1,421	1,262
	N50 (bp)	1,843	1,728	1,931	1,754

### Characterization of Microsatellite in *P. chienii* Transcriptome

**FIGURE 1:**
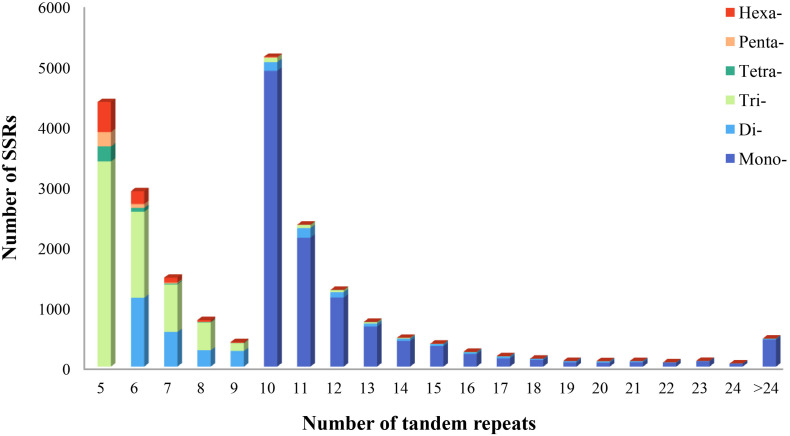
Distribution of microsatellite motif types and tandem repeat numbers in *P. chienii* transcriptome.

Using the MISA program, a total of 21,835 potential microsatellite loci were identified from 161,131 non-redundant unigenes, with 2,969 unigenes containing more than one microsatellite loci. The frequency of microsatellite in *P. chienii* transcriptome was 13.55%; an average of one microsatellite loci was found every 9.18 kb (Table 3). The most abundant repeat motif types were mono-nucleotide (10,930, 50.06%), which accounted for about half of total SSRs, followed by tri-nucleotide (6,418, 29.39%), di-nucleotide (2,946, 13.49%), hexa-nucleotide (844, 3.87%), tetra-nucleotide (354, 1.62%), and penta-nucleotide (343, 1.57%) (Table 3). The number of tandem repeats of microsatellite motifs ranged from five to 107. Microsatellite with ten tandem repeats (5,141, 23.54%) were the most common, followed by five tandem repeats (4,393, 20.12%), six tandem repeats (2,913, 13.34%), and 11 tandem repeats (2,357, 10.79%). Microsatellite motifs with > 24 tandem repeats only accounted for 2.13% (Figure 1).

**TABLE 3:** Analysis results of microsatellite based on the *P. chienii* transcriptome.

Items	Number
Total number of sequences examined	161,131
Total size of examined sequences (bp)	200,495,148
Total number of identified microsatellite loci	21,835
Number of microsatellites containing sequences	17,974
Number of sequences containing more than 1 microsatellite loci	2,969
Number of microsatellites present in compound formation	1,513
Frequency of microsatellite loci	13.55
Distribution density of microsatellite loci (kb)	9.18
Mono-nucleotide	10,930
Di-nucleotide	2,946
Tri-nucleotide	6,418
Tetra-nucleotide	354
Penta-nucleotide	343
Hexa-nucleotide	844

**FIGURE 2:**
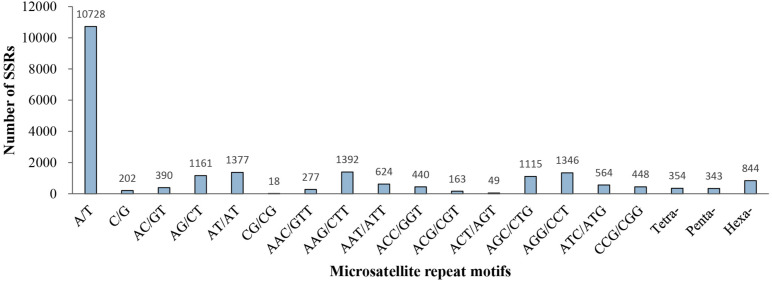
Distribution of microsatellite repeat motifs in *P. chienii* transcriptome.

Of the 21,835 microsatellite loci, 239 different repeat motifs were detected. For the mono-nucleotide motif, A/T was the most abundant type with a frequency of 49.13%. For the di-nucleotide repeat motifs, AT/AT (6.31%) and AG/CT (5.32%) were the dominant types. The two most frequent types in the tri-nucleotide were AAG/CTT (6.38%) and AGG/CCT (6.16%). Moreover, a low percentage (7.06%) of tetra-, penta- and hexa-nucleotide repeat motifs were observed in all identified microsatellite motifs (Figure 2).

Microsatellite length ranged from 10 to 107 bp, with an average length of 16.23 bp. In general, the length of microsatellites differed significantly between the motif size classes (Kruskal–Wallis rank sum test, *P* < 2.2e^–16^), and their average length decreased with the increase of the motif size (Nemenyi test, *P* < 2.2e^–16^). However, there was no significant difference in length between tetra- and penta-nucleotide according to the pairwise length comparisons by the Nemenyi test (Supplementary Figure 1).

### Microsatellite Distribution in Different Genic Regions

**FIGURE 3:**
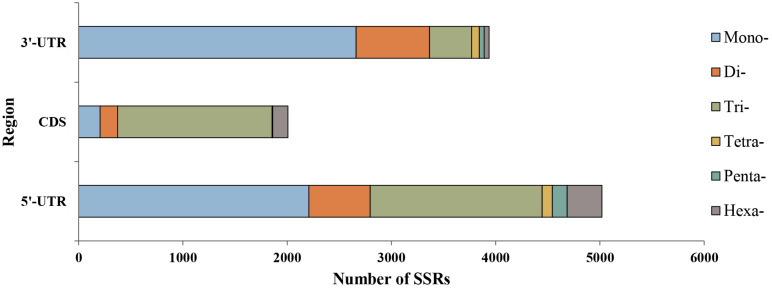
Distribution of six microsatellite repeat types in different genic regions of *P. chienii*.

We explored the distribution characteristics of microsatellite loci in *P. chienii* transcriptome. Of the 21,835 microsatellite loci, 2,007 and 8,958 were located in the coding sequence regions (CDS) and untranslated regions (UTRs), respectively (Figure 3). The remaining 10,870 microsatellites were excluded from the analysis because there was insufficient information to determine their distribution. Microsatellites in different genic regions (CDS, 5′-UTRs, and 3′-UTRs) showed distinct patterns of distribution (χ^2^ = 2993.2, *P* < 2.2e^–16^). CDS contained fewer microsatellites and was dominated by tri-nucleotides (1,481, 73.79%), whereas the mono- and di-nucleotide microsatellites in the UTRs (4,869, 54.35%) were abundant. Smaller motif types (mono-, di-, and tri-nucleotide microsatellites) were also observed to be more common in the transcriptome (Figure 3). Furthermore, significant differences in the length of microsatellite among three regions (CDS, 5′-UTR, and 3′-UTR) (Kruskal–Wallis rank sum test, *P* < 2.2e^–16^, Supplementary Figure 2). The average length of microsatellites located in the CDS regions (17.84 bp) was slightly longer than in the UTRs (16.43 bp). The length of the microsatellite was also affected by the interaction of the regions and motif types according to the Kruskal–Wallis rank sum test (*P* < 2.2e^–16^, Supplementary Figure 3).

### Functional Annotation and Classification of Unigenes Containing Microsatellites

To predict the potential functions of microsatellites, all microsatellite-containing unigenes were blasted against the public databases using the BLASTX program. As a result, 12,652 (70.39%) unigenes were annotated in Nr, 6,660 (37.05%) in Nt, 10,622 (59.10%) in Swiss-Prot, 4,274 (23.78%) in KOG, 11,267 (62.68%) in Pfam, 11,334 (63.06%) in GO, and 5,417 (30.14%) in KEGG. A total of 2,045 (11.38%) unigenes were annotated in all seven databases, and 14,264 (79.36%) unigenes were annotated in at least one database (Table 4).

**TABLE 4:** Summary of functional annotation results of unigenes containing microsatellite in *P. chienii* transcriptome.

Annotated databases	Number of unigenes	Percentage (%)
Nr	12,652	70.39
Nt	6,660	37.05
Swiss-Prot	10,622	59.10
KOG	4,274	23.78
Pfam	11,267	62.68
GO	11,334	63.06
KEGG	5,417	30.14
Annotated in all databases	2,045	11.38
Annotated in at least one database	14,264	79.36
Total	17,974	100

**FIGURE 4:**
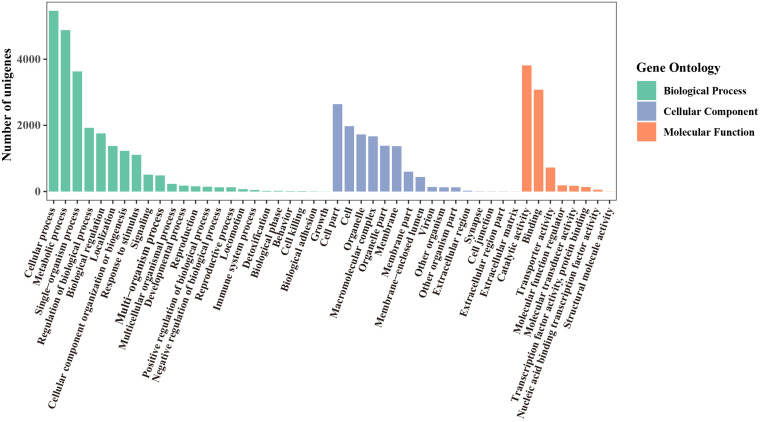
GO functional classification of unigenes containing microsatellite in *P. chienii* transcriptome.

Using the Blast2GO Program, the microsatellite-containing unigenes were divided into three categories: biological process (BP), cellular component (CC), and molecular function (MF), including 48 sub-categories (Figure 4). In the BP category, “cellular process” (5,464, 48.21%) and “metabolic process” (4,879, 43.05%) were represented prominently. Within the CC category, “cell” (1,975, 17.43%) and “cell part” (2,642, 23.31%) were the most abundant terms, whereas only a few unigenes were assigned to “extracellular region part” (3), “cell junction” (3), and “extracellular matrix” (2). Among eight different MF categories, “catalytic activity” (3,813, 33.64%) and “binding” (3,078, 27.16%) were the two most frequent classes. These results indicated that the SSRs-containing unigenes were mainly involved in the basal metabolism of cells.

**FIGURE 5:**
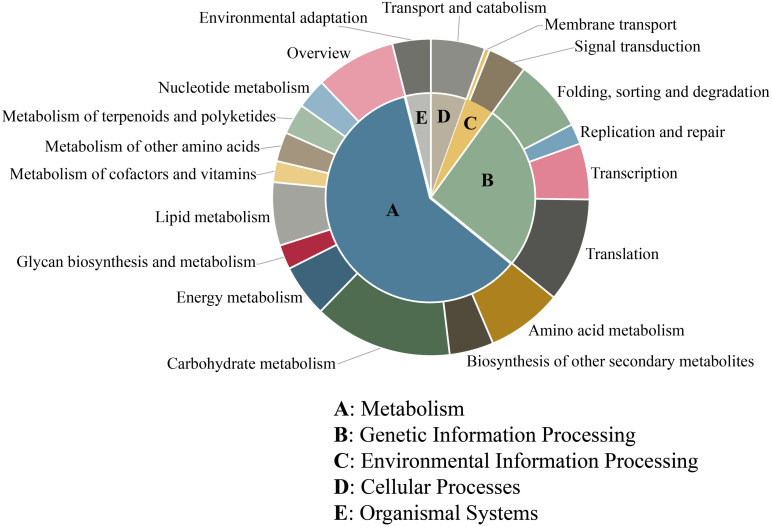
KEGG pathway classification of unigenes containing microsatellite in *P. chienii* transcriptome.

Furthermore, KEGG analysis was used to determine the biological pathways and functions of the unigenes containing microsatellites. In total, 5,125 (28.51%) unigenes were mapped in 124 different pathways and classified into 5 main categories. The largest category was “metabolism” (3,086, 56.97%), followed by “genetic information processing” (1,326, 24.48%), “cellular processes” (284, 5.24%), “environmental information processing” (228, 4.21%), and “organismal systems” (201, 3.71%) (Figure 5). Among these 124 pathways, the most represented pathways including “spliceosome,” “ribosome,” “carbon metabolism,” “plant–pathogen interaction,” “plant hormone signal transduction,” “biosynthesis of amino acids,” “RNA transport,” “protein processing in endoplasmic reticulum,” “starch and sucrose metabolism,” and “ubiquitin mediated proteolysis” (Supplementary Table 2).

**FIGURE 6:**
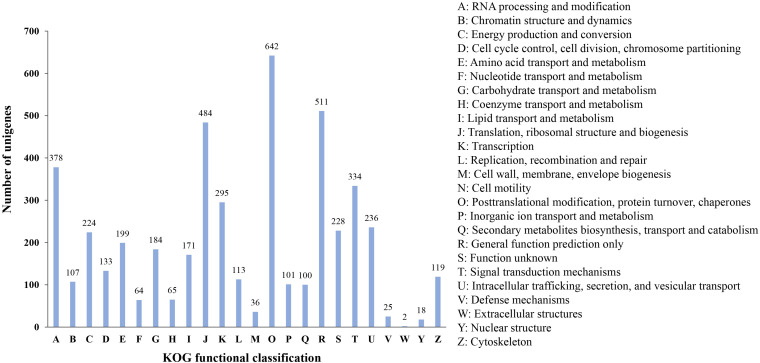
KOG functional classification of unigenes containing microsatellite in *P. chienii* transcriptome.

Finally, all microsatellite-containing unigenes were searched against the KOG database to classify and functionally annotation the orthologous gene products. The results showed that a total of 4,274 unigenes were classified into 24 functional groups. Among them, the three largest groups were “posttranslational modification, protein turnover, chaperones” (642, 15.02%), “general function prediction only” (511, 11.96%), and “translation, ribosomal structure and biogenesis” (484, 11.32%), respectively. However, the smallest categories were “extracellular structures” and “nuclear structure,” with two and 18 genes annotated, respectively (Figure 6). These results reflected that the unigenes containing microsatellites had extensive basic biological functions.

### GO and KEGG Enrichment Analysis of the Microsatellite-Containing Unigenes

**FIGURE 7:**
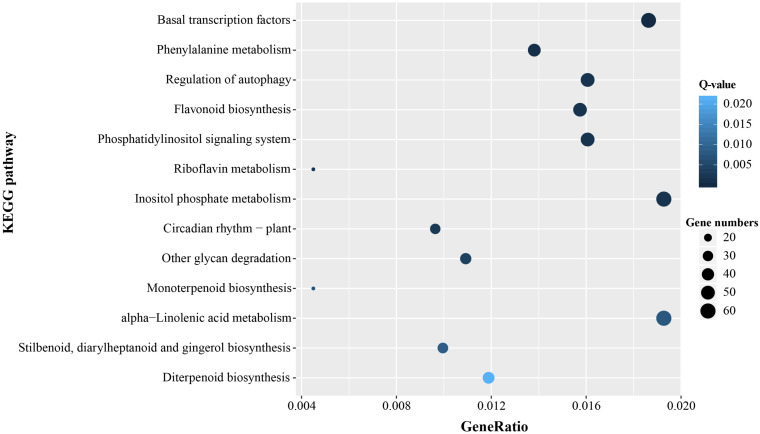
Significant enrichment KEGG pathways of unigenes containing microsatellite.

In order to further identify molecular and biological functions of the unigenes containing microsatellites, Fisher’s exact test was used for GO and KEGG enrichment analysis, respectively. There was significant enrichment of microsatellites in a total of 211 GO terms and most were mapped in transcription, regulation of transcription, and binding (*Q*-value < 0.05, Supplementary Table 3). KEGG pathway enrichment analysis indicated that the microsatellite-containing unigenes showed significantly enriched in 13 pathways (*Q*-value < 0.05), and Nine of them are related to basal metabolism and biosynthesis of cells. Notably, the remaining four pathways involved in gene expression regulation, and might play important roles in the response to stress (Figure 7).

### Development and Validation of Microsatellite Markers

Based on the SSR-containing unigene sequences, 375 primer pairs were randomly selected and synthesized for the amplification and validation of microsatellite markers. After screening, 77 primer pairs (20.59%) successfully amplified and produced clear bands with expected size, 40 pairs of primers were identified as polymorphic, and 37 pairs were monomorphic (Supplementary Table 4). Finally, 20 primers with high polymorphism were selected and used for genetic diversity analysis (Table 5). In total, 128 alleles were detected at the 20 polymorphic microsatellite loci. The number of amplified alleles per locus (Na) ranged from 2 (*P. chienii*-198) to 16 (*P. chienii*-152), with an average of 6.4 alleles per locus. The observed (Ho) and expected (He) heterozygosity ranged from 0.000 to 1.000 and from 0.129 to 0.887, with an average of 0.349 and 0.527, respectively. The PIC values ranged from 0.125 to 0.877, with an average of 0.483. Besides, a high frequency of null alleles was detected at six loci (*P. chienii*-17, *P. chienii*-25, *P. chienii*-86, *P. chienii*-162, *P. chienii*-267, and *P. chienii*-288), and one pair of loci (*P. chienii*-28 and *P. chienii*-162) showed LD. HWE test revealed that 13 of 20 microsatellite loci showed significant deviations from the equilibrium after Bonferroni correction (*P* < 0.05) (Table 5).

**TABLE 5:** Characteristics of 20 newly developed polymorphic microsatellite markers in *P. chienii*.

Locus	Primer sequence (5′-3′)	Repeat motif	Size range (bp)	Annealing temperature (°C)	Na	Ho	He	PIC*	Null	GenBank accession no.
*P. chienii*-17	F: GGTCGAGTACGTGGTGGTTT R: GCCTGCGCTGTCATAAACTG	(AGG)5	158–161	57	3.000	0.145	0.257	0.237**	0.2703 (Y)	MT563347
*P. chienii*-20	F: TGTGCCAGTACTGCTACTGC R: TGAATGCGTGCGGAAACAAG	(ACC)6	183–195	57	5.000	0.209	0.585	0.517	−0.0267 (N)	MT563350
*P. chienii*-25	F: GATGCCGCTGGTTTCAATCC R: GCCGTACCGATTGGGATCAT	(GGA)8	198–214	57	5.000	0.309	0.397	0.366*	0.315 (Y)	MT563351
*P. chienii*-28	F: GAGTGGGAGACGAAGAGTGC R: CGAAGTGGGCTGCAACAATG	(CTC)8	261–266	57	6.000	0.600	0.670	0.626	0.0196 (N)	MT563352
*P. chienii*-29	F: AGCTGCAAGGCTACACAGAG R: CAATCCCGGGCCTGTTAGAA	(GAA)5	235–239	57	5.000	0.318	0.563	0.500**	−0.1413 (N)	MT563353
*P. chienii*-36	F: GGGCCATCCTCTTCCTCAAC R: CTCGACACTGCTCCACATCT	(TCC)8	240–247	57	3.000	0.055	0.129	0.125**	0.0299 (N)	MT563358
*P. chienii*-75	F: CGCTCCAACGAATCCAACC R: TAATGCCATCCGCACAACC	(CAGAAG)5	241–277	57	14.000	0.743	0.844	0.826	−0.1586 (N)	MT563368
*P. chienii*-86	F: GAATTTGAAGCACGGCCTCA R: GAGTGCCCTGCTTTCTGGAT	(GGCACC)5	242–277	57	7.000	0.156	0.595	0.549**	0.3533 (Y)	MT563369
*P. chienii*-134	F: ACGCCACGTTAGGACACAAT R: CCTAGATCAAGAGCGGCCTG	(CTT)6	238–319	57	6.000	0.227	0.389	0.364	0.0983 (N)	MT563374
*P. chienii*-141	F: CTGTCAACAAGCGGCTTTCC R: AGAGCCGGGGGAAAATTGAG	(CGG)7	232–319	57	8.000	0.473	0.694	0.640**	0.0296 (N)	MT563377
*P. chienii*-152	F: CCCATCTGAACCCACGCTAA R: AAAGCGCTCATGCCCAAAAC	(GGC)7	239–300	57	16.000	1.000	0.887	0.877**	−0.2134 (N)	MT563378
*P. chienii*-162	F: ACCTATCACCTCCTCGACCC R: CCGTTCCATCACTGTGGACA	(CCACCG)6	203–229	55	13.000	0.573	0.807	0.784**	0.3871 (Y)	MT563379
*P. chienii*-198	F: GAGGGATACAGAAGCACAG R: TATGACAAACCCAAACGAG	(ATA)5	276–288	56	2.000	0.591	0.416	0.330**	0 (N)	MT563329
*P. chienii*-214	F: GACAACGGCAAAGGAGGAAT R: GCGATAGCCACCAAAGACAT	(ATA)6	319–322	58	3.000	0.000	0.329	0.290**	0 (N)	MT563334
*P. chienii*-216	F: TGCGGTTCAGTAACAGTCCTTC R: TCCCCCACCTCTTCCCAG	(CTCCTG)5	439–452	58	6.000	0.657	0.513	0.408	−0.314 (N)	MT563335
*P. chienii*-233	F: TGTGTGAAAGGACAAGGCGT R: GCACCCTATTCACCCGAGAT	(ATGCAG)7	225–259	56	5.000	0.318	0.474	0.431	−0.0267 (N)	MT563337
*P. chienii*-267	F: CCCCTCATTGACAGGTTC R: AAGATAGTCGGGACACCAAG	(CTG)5	309–320	56	6.000	0.036	0.336	0.306**	0.3232 (Y)	MT563381
*P. chienii*-288	F: CACGCCCACCATAGTTGT R: GGAGGAAGATGTCGTTGAAG	(AAGG)5	263–270	58	3.000	0.045	0.523	0.449**	0.2884 (Y)	MT563386
*P. chienii*-341	F: GACCTCTTACCAGCTGCGAG R: ACCACCGGTTTCAGTTTCGT	(CCT)11	208–227	62	9.000	0.200	0.765	0.726**	0 (N)	MT563390
*P. chienii-*358	F: TAAGTGGCTGCTGCATCACA R: TACAGCAGCAGCAGAGCTTT	(TCC)5	249-251	58	3.000	0.318	0.360	0.302	0.1828 (N)	MT563392

Locus, locus name; Na, number of alleles; Ho, observed heterozygosity; He, expected heterozygosity; PIC, Polymorphism information content (*P < 0.05, significantly departures from Hardy–Weinberg equilibrium; **P < 0.01, extremely significantly departures from Hardy–Weinberg equilibrium); Null, the frequency of null allele (Y represents that the SSR locus may have a null allele, N represents that the SSR locus may not have null allele).

At the population level, MS population had the highest level of genetic diversity (Na = 3.450, Ne = 1.997, Ho = 0.384, and He = 0.389), whereas the lowest diversity levels were in ZJJ population (Na = 2.850, Ne = 1.759, Ho = 0.272, and He = 0.340). All of the inbreeding coefficient (*F*_IS_) values were positive for four populations, which indicated that there was a certain degree of inbreeding in *P. chienii* populations (Table 6). Besides, bottleneck analysis showed that YS and BJS populations showed heterozygous excess and might experience a significant genetic bottleneck under the SMM models (*P* < 0.05) (Supplementary Table 5).

**TABLE 6:** Genetic diversity parameters of four populations of *P. chienii*.

Population	N	Na	Ne	I	Ho	He	PPB (%)	*F*_IS_
MS	31	3.450	1.997	0.702	0.384	0.389	90	0.031^*ns*^
YS	30	3.500	1.918	0.670	0.360	0.357	85	0.008^*ns*^
BJS	30	3.650	2.033	0.716	0.350	0.381	100	0.099**
ZJJ	19	2.850	1.759	0.602	0.272	0.340	85	0.227**
Species level	110	6.400	2.814	1.071	0.349	0.527	100	0.342

N, numbers of individuals; Na, number of alleles; Ne, number of effective alleles; Ho, observed heterozygosity; He, expected heterozygosity; I, Shannon’s information index; PPB, percentage of polymorphic loci; F_IS_, inbreeding coefficient. ^ns^P > 0.05, **P < 0.01.

### Transferability of Microsatellite Markers

To evaluate the transferability of the microsatellite markers from the *P. chienii* transcriptome, we performed cross-species amplification analysis for these 20 novel microsatellite markers in *A. argotaenia*, 14 primers were amplified products. Therefore, the overall transferability rates were 70%. Among the 14 primer pairs, 12 showed polymorphisms in 58 *A. argotaenia* samples. For the 12 polymorphic loci, there were 2–7 alleles at each locus, with a total of 50 alleles. The Ho, He, I, and PIC per locus ranged from 0 to 1.000, 0.133 to 0.741, 0.257 to 1.539, and 0.155 to 0.664, respectively (Supplementary Table 6). The microsatellite loci were moderately polymorphic.

## Discussion

### Characterization of Microsatellites in *P. chienii* Transcriptome

Recently, next-generation sequencing (NGS) technologies, such as transcriptome sequencing, have made it possible to develop large-scale microsatellite markers (Taheri et al., 2018). In this study, a total of 21,835 microsatellites were identified from 161,131 unigenes of the transcriptome. The frequency of microsatellites was 13.55%, and the distribution density was one SSR per 9.18 kb, which was much higher than previous reports in *Pinus dabeshanensis* (1/23.08 kb) (Xiang et al., 2015) and *Larix principis-rupprechtii* Mayr (1/26.8 kb) (Dong et al., 2018), but lower than *Cephalotaxus hainanensis* (1/1.652 kb) (Qiao et al., 2014) and *Glyptostrobus pensilis* (1/7.59 kb) (Li et al., 2019). This result showed that SSR sites were abundant in *P. chienii* transcriptome. The frequency and density of SSRs varied from species to species, which could be attributed to the size of unigene assembly dataset, the search criteria, mining tools for SSR locus, and genome organization (Morgante et al., 2002; Varshney et al., 2005; Taheri et al., 2019).

In our study, six different repeat motifs were identified. Excluding the mono-nucleotide repeats, tri-nucleotide (29.39%) repeats were the most abundant type, followed by di-nucleotide repeats (13.49%). The AAG/CTT (1,382, 6.38%) motif was the most abundant of the tri-nucleotide repeats, followed by AGG/CCT (1,346, 6.16%). This result might be common in conifer species, including *Glyptostrobus pensilis* (Li et al., 2019), *Larix principis-rupprechtii* Mayr (Dong et al., 2018), and *Torreya grandis* (Zeng et al., 2018). Among the di-nucleotide repeats, AT/AT (1377, 6.31%) was the most dominant motif, which was consistent with studies in *Cephalotaxus hainanensis* (Qiao et al., 2014) and *Pinus koraiensis* (Li et al., 2020), but not in *Pennisetum purpureum* (Zhou et al., 2018), *Liquidambar formosana* (Chen et al., 2020), and *Rhododendron arboretum* (Sharma et al., 2020). By contrast, the lowest frequency of CG/CG motifs (18, 0.08%) of the di-nucleotide repeats, which might be explained by cytosine methylation inhibited gene transcription (Chen et al., 2015; Xing et al., 2017).

The length of the microsatellite was one of the main factors affecting its polymorphism. According to Temnykh et al. (2001), SSR polymorphism can be considered low, medium or high if length < 12 bp, 12 ≤ length < 20 bp, and length > 20 bp. In this study, SSR length between 12 and 20 bp (10638, 48.72%) accounted for the largest proportion. Therefore, it can be speculated that the microsatellites in *P. chienii* transcriptome may have a moderate level of polymorphism. In addition, the mutation rate of microsatellite is positively correlated with its length (Calabrese and Sainudiin, 2005). In *P. chienii*, six microsatellite motifs were found to have significant differences in length (Kruskal–Wallis rank sum test, *P* < 2.2e^–16^), and decreased with increasing motif size (Nemenyi test, *P* < 2.2e^–16^). Longer microsatellites were expected to have a high mutation rate because of more chances of replication slippage (Calabrese and Sainudiin, 2005). Therefore, tetra-, penta-, and hexa-nucleotide microsatellites might have higher mutation rates than those of the mono-, di-, and tri-nucleotide microsatellites.

### Microsatellite Distribution in Different Genic Regions

Studies have shown that microsatellites are non-randomly distributed across protein-coding regions, UTRs, and introns (Li et al., 2004). In this study, the number of microsatellites in UTRs was much higher than that in CDS regions. One possible reason was that microsatellites located in UTRs were subject to less selection pressure and evolutionary constraints, thus, the microsatellites located in this region was more prone to expansion. In contrast, due to the high selection pressure of the microsatellite in coding regions, the structure and function of the gene will be seriously damaged if “indel” mutation occurs in this region (Levinson and Gutman, 1987; Li et al., 2004). We also found that the microsatellites located in CDS regions were dominated by tri-nucleotides, while other SSR motifs accounted for a small proportion. This was common in many plants, such as *Phoenix dactylifera* L. (Zhao et al., 2013), *Tetraena mongolica* (Dang et al., 2020), and *Paeonia lactiflora* (Wan et al., 2020). Compared to other repeat types, tri-nucleotide is less likely to frame-shift mutations in coding regions (Metzgar et al., 2000).

Although some studies have shown that the microsatellites in coding regions were subject to higher evolutionary constraints and thus exhibited a shorter length. For example, Liu et al. (2016) found in a study on *Sargassum thunbergii* that microsatellites in UTR regions were much longer than those in CDS regions. However, our results showed that the microsatellites located in UTR regions had relatively short average length, which could be attributed to the large number of mono- and di-nucleotide microsatellites located in these regions.

### Potential Function of Unigenes Containing Microsatellites

Microsatellites derived from the transcribed sequence might be directly related to gene function and thus might play an adaptive and evolutionary role in affecting gene products, inducing phenotypic changes, and regulating gene expression (Li et al., 2004). In order to reveal the potential functions of microsatellites-containing unigene sequences, functional annotation and classification of these unigenes were carried out. GO functional annotations showed that a large number of unigenes containing microsatellites were assigned to “cellular process,” “metabolic process,” “cell,” “cell part,” “catalytic activity,” and “binding” terms, suggesting they might be related to the basal metabolism and life activities of *P. chienii*. Similarly, KEGG and KOG analysis indicated that SSRs-containing unigenes had a wide range of biological functions and were involved in various aspects of *P. chienii* growth and development. In addition, GO enrichment analysis showed that unigenes containing microsatellites were significantly enriched in terms related to “transcription,” similar to the reports in *Onobrychis viciifolia* (Shen et al., 2019) and Siberian wildrye (Zhou et al., 2016). Additionally, we found that some unigenes containing microsatellites were associated with transcription factors. TFs (Transcription Factors) regulate a lot of biological processes, including plant defense, response to a range of abiotic and biotic stresses, and tolerance to disease and environmental stress (Singh et al., 2002). As an ancient relic plant, *P. chienii* might be disturbed by various adverse factors during the long evolutionary process. However, it still survived to the present day and has strong adaptability to the environment. From the point of view of molecular biology, the microsatellites identified in this study that are related to transcription factors might serve as important regulatory buttons for gene expression in the adaptive evolution of *P. chienii*. Likewise, KEGG enrichment analysis revealed that four pathways that were associated with gene expression regulation (ko03022, ko04140, ko04070, and ko04712) showed significant enrichment, indicating that they played an important role in the response to environmental stress.

Additionally, there may be some differences in the functions of microsatellites in different tissues. However, we only analyzed the distribution and function of microsatellites in *P. chienii* transcriptome as a whole. Ince et al. (2010b) studied microsatellite-containing gene expression profiles in 16 different tissues and at different developmental stages in pepper (*Capsicum annuum* L.), and found that tissue-specific genes contained more dinucleotide microsatellites, and housekeeping genes contained more trinucleotide microsatellites. Therefore, we can further compare the expression patterns of microsatellite-containing genes in four tissues of *P. chienii* in the future, so as to deepen the understanding of tissue-specific expression of microsatellites.

### Validation of Microsatellite Markers

The 375 pairs of primers were randomly selected for validating the amplification quality and polymorphisms of the microsatellites in the present study. Among of them, only 77 (20.53%) produced clear bands with expected size. This low amplification success rate (20.53%) was comparable to that reported in some conifer species, such as *Abies alba* (24%) (Postolache et al., 2014), *Torreya grandis* (29.25%) (Zeng et al., 2018), and *Pinus koraiensis* (32%) (Li et al., 2020). There were two main reasons for the low success rate of amplification in *P. chienii*. On the one hand, there were a large number of pseudogenes in conifer genomes, which may hinder the amplification of microsatellite markers. On the other hand, the highly repetitive sequences in conifer genomes, such as transposons, might affect the amplification efficiency of microsatellites (Kovach et al., 2010). Besides, the presence of large introns or indels, assembly errors in the unigenes, and low specificity might cause the primer amplification failure (Bazzo et al., 2018; Niu et al., 2019).

### Genetic Diversity of *P. chienii* Populations

Genetic diversity is the product of long-term evolution of a species or population and is a necessary condition for the survival and sustainable development of populations (Zhang and Zhou, 2013). We validated the polymorphism of the 20 polymorphic microsatellite markers using four populations of *P. chienii*. A total of 128 alleles were detected at the 20 loci. Among of them, loci *P. chienii*-152 (with 16 alleles) and *P. chienii*-75 (with 14 alleles) showed a high level of polymorphism. Loci *P. chienii*-152 was highly homologous to an unknown protein of *Picea sitchensis*. Loci *P. chienii*-75 was associated with a gene encoding R2R3-MYB transcription factor, therefore, played an important regulatory role in *P. chienii* growth and development, as well as in response to abiotic and biotic stress. At the species level, there was a moderate level of genetic diversity (Na = 6.4, Ho = 0.349, He = 0.527, I = 1.071) for *P. chienii*, which was similar to the previous reports of Kou et al. (2020) using nuclear loci (*H*_d_ = 0.5400, π = 0.00265), but higher than that of Su et al. (2009) by ISSR markers (*h* = 0.2118, *I* = 0.2390). In addition, compared with other conifer species such as *Larix principis-rupprechtii* Mayr (Na = 3.850, Ho = 0.487, He = 0.490) (Dong et al., 2018) and *Pinus koraiensis* (Na = 6.45, Ho = 0.299, He = 0.311) (Li et al., 2020), *P. chienii* has a slightly higher level of genetic diversity (Na = 6.4, Ho = 0.349, He = 0.527). The abundance of genetic variation at the species level can be attributed to internal and historical factors. As an ancient tertiary relic plant, *P. chienii* has accumulated a large number of genetic variations during the long-term evolution, so it is expected to maintain a high level of genetic diversity. Besides, the Central and Nanling regions of China were less affected by the Quaternary glacial and became the possible refugia of many relic plants (Chen et al., 2011). We speculated that these refugia might be beneficial to the preservation of genetic variation in *P. chienii*. At the population level, all the four populations of *P. chienii* showed a low level of genetic diversity, which could be explained by its population size and distribution characteristics. The wild populations of *P. chienii* were few and scattered distributions, which limited the gene flow among populations and reduced genetic diversity. In addition, the small population size might result in the bias of genetic diversity in this study. We will sample more individuals of *P. chienii* to further explore its genetic variation in the future.

### Transferability of Microsatellite Markers

In general, microsatellite markers developed from transcriptome data/ESTs showed a higher level of transferability in related species (Ince et al., 2010a, 2014; Yan et al., 2017). In the present study, 14 microsatellite markers could successfully amplify in *A. argotaenia*, and 12 of them were polymorphic in all *A. argotaenia* individuals. The transferability ratio was 70%, which was higher than the reported in *Capsicum* (32.11%) (Ince et al., 2010c), and *Elymus* (49.11%) (Zhou et al., 2016), indicating that the markers developed in the present study had good potential for cross-species amplification and could be used in future genetic studies of other Taxaceae species.

## Conclusion

In this study, we identified and characterized the frequency, distribution, and function of microsatellites based on transcriptome data of *P. chienii*. The unigenes containing microsatellites had a wide range of biological functions, most of which were associated with basic metabolism, and a few might be involved in expression regulation of gene and response to environmental stress. These results provided new insights into the role of microsatellites in the transcriptome. In addition, twenty microsatellite markers were developed for *P. chienii* with high polymorphism and good transferability, which will facilitate the genetic study in *P. chienii* and other related species.

## Data Availability Statement

The datasets presented in this study can be found in online repositories. The names of the repository/repositories and accession number(s) can be found below: https://www. ncbi.nlm.nih.gov/genbank/, SRR11715801; https://www.ncbi. nlm.nih.gov/genbank/, SRR11715800; https://www.ncbi.nlm.nih.gov/genbank/, SRR11715799; https://www.ncbi.nlm.nih.gov/genbank/, SRR11715798. The EST sequences in this study have been submitted to GenBank with accession numbers MT563329–MT563392.

## Author Contributions

YS and TW conceived and designed the study and revised the manuscript. RX and ZW performed the experiments, analyzed the data, and wrote the manuscript. All the authors contributed to the article and approved the submitted version.

## Conflict of Interest

The authors declare that the research was conducted in the absence of any commercial or financial relationships that could be construed as a potential conflict of interest.

## Footnotes

1 http://pgrc.ipk-gatersleben.de/misa/

## Supplementary Material

Supplementary Figure 1 Box plots of the length distribution of six microsatellite repeat motifs.

Supplementary Figure 2 Box plots of the length distribution of microsatellite loci located in different genic regions.

Supplementary Figure 3 Box plots of the length distribution of six microsatellite repeat motifs in different genic regions.

Supplementary Table 1 Sampling location information of four populations of *A. argotaenia*.

Supplementary Table 2 124 KEGG pathways annotated for the unigenes containing microsatellites.

Supplementary Table 3 The results GO enrichment analysis for unigenes containing microsatellites.

Supplementary Table 4 The 77 primers pairs of *P. chienii* screened in this study.

Supplementary Table 5 Bottleneck analysis for four populations of *P. chienii*.

Supplementary Table 6 PCR amplification results of 12 pairs of polymorphic SSR primers in *Amentotaxus argotaenia*.
