# Supplementary figures and images for: Characterization and Development of Microsatellite Markers in Pseudotaxus chienii (Taxaceae) Based on Transcriptome Sequencing
Source: Front Genet. 2020 Oct 15;11:574304. doi: 10.3389/fgene.2020.574304 (PMC7593448; doi:10.3389/fgene.2020.574304)

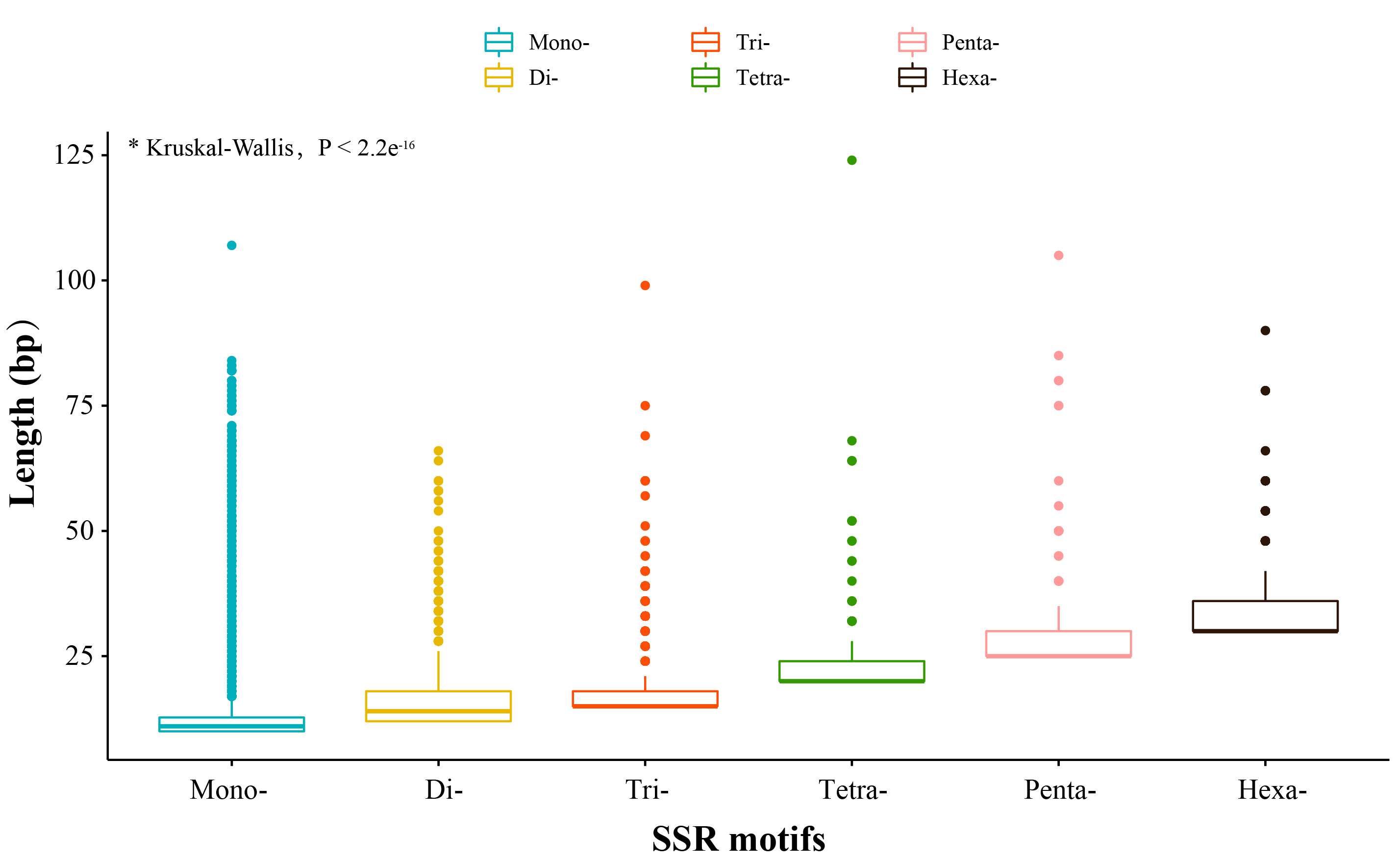

Supplement: Supplementary Figure 1 — Box plots of the length distribution of six microsatellite repeat motifs. [file Image_1.JPEG]

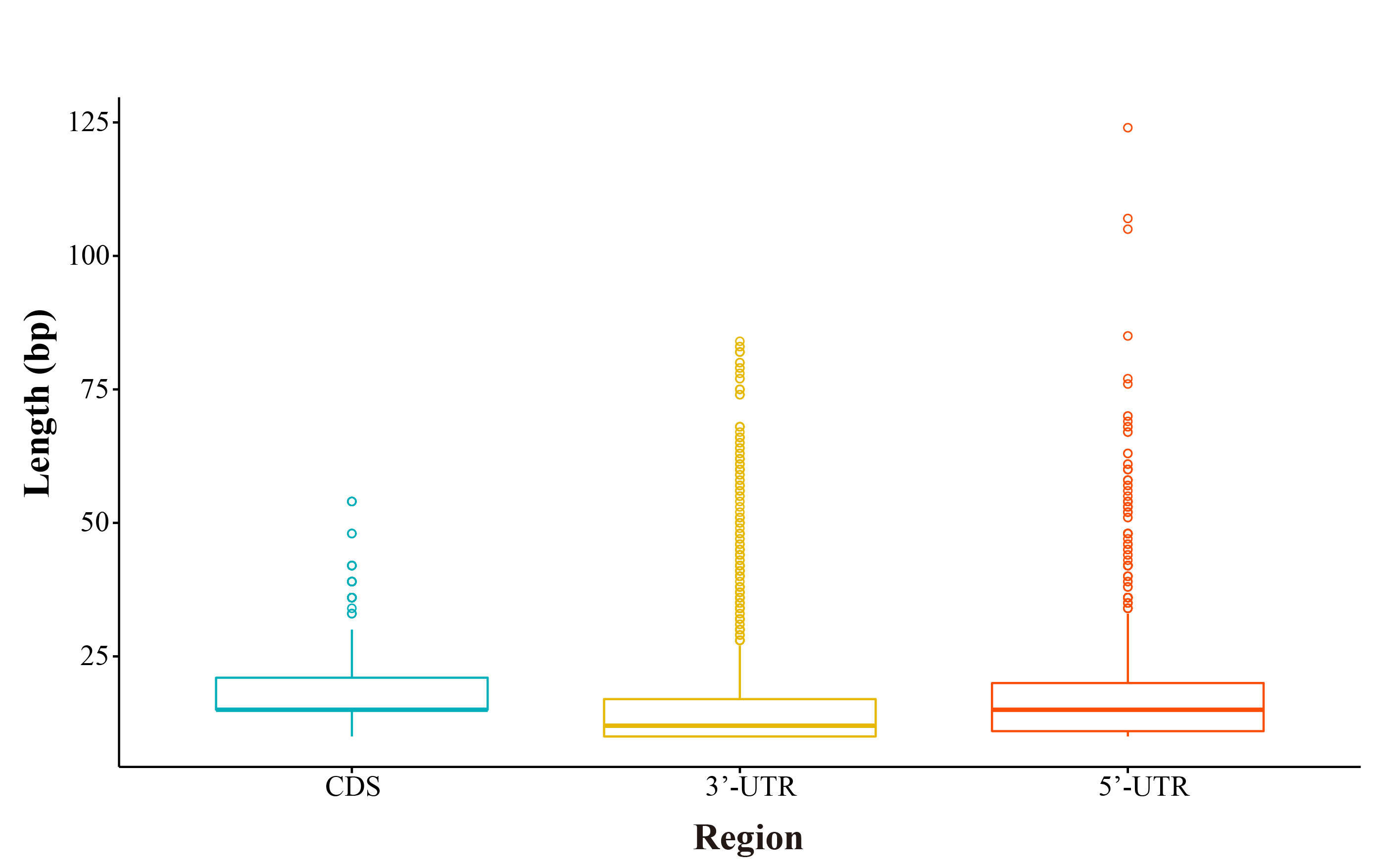

Supplement: Supplementary Figure 2 — Box plots of the length distribution of microsatellite loci located in different genic regions. [file Image_2.JPEG]

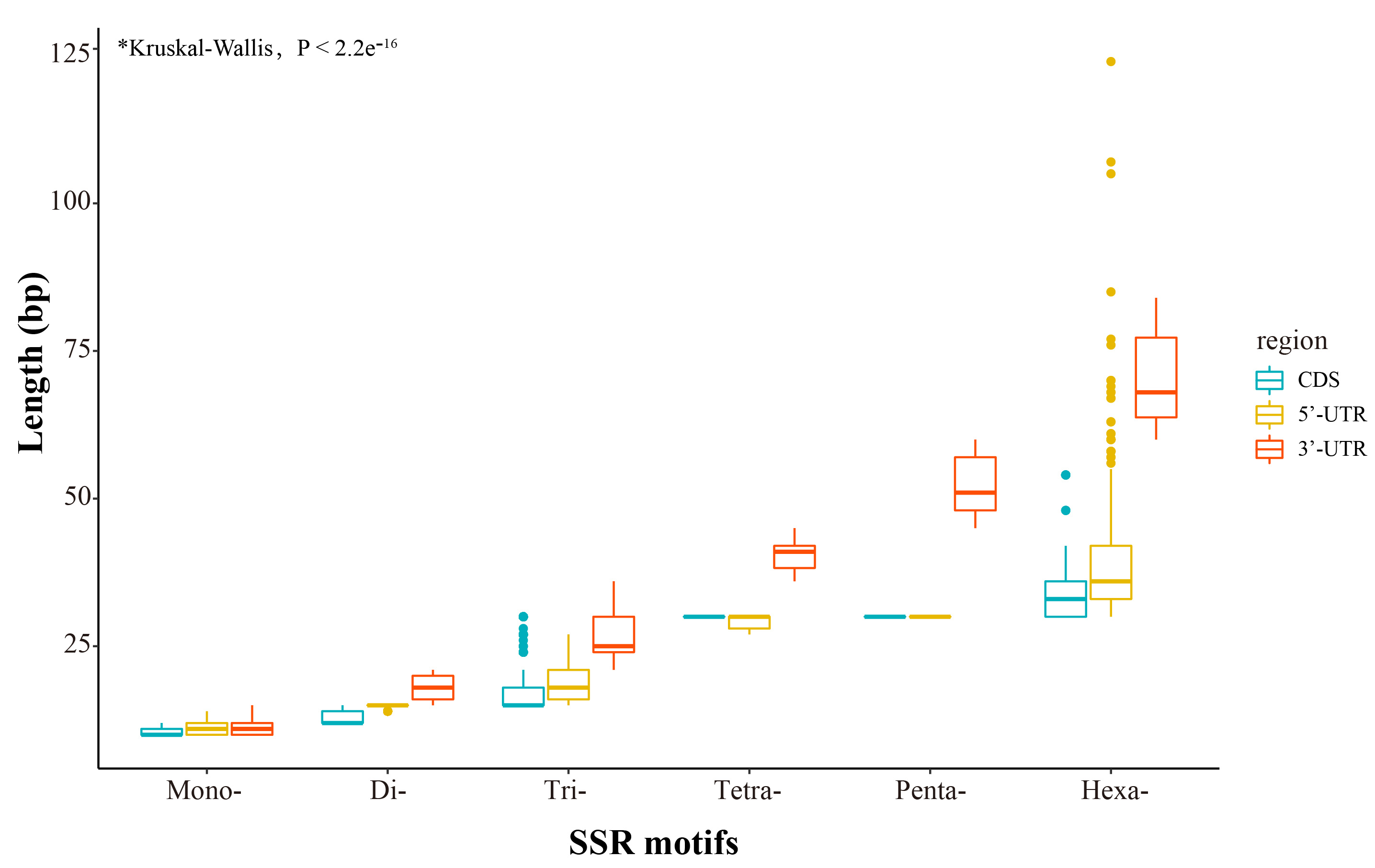

Supplement: Supplementary Figure 3 — Box plots of the length distribution of six microsatellite repeat motifs in different genic regions. [file Image_3.JPEG]
