# Supplementary material for: Characterization and Development of Microsatellite Markers in Pseudotaxus chienii (Taxaceae) Based on Transcriptome Sequencing
Source: Front Genet. 2020 Oct 15;11:574304. doi: 10.3389/fgene.2020.574304 (PMC7593448; doi:10.3389/fgene.2020.574304)
Supplement: Supplementary Table 1 — Sampling location information of four populations of A. argotaenia. [file Table_1.DOCX]

Supplementary Table 1 Sampling location information of four populations of *A. argotaenia.*

| Population | Location | Sample  Size | Latitude  (N) | Longitude  (E) |
| --- | --- | --- | --- | --- |
| JQS | Jiuqushui, Hunan | 23 | 26°34ʹ02.10ʹʹ | 114°04ʹ42.27ʹʹ |
| CP | Chuanping, Jiangxi | 9 | 26°45ʹ26.36ʹʹ | 114°10ʹ12.63ʹʹ |
| WGS | Wugongshan, Jiangxi | 21 | 27°27ʹ53.10ʹʹ | 114°09ʹ56.63ʹʹ |
| QNS | Qiniangshan, Guangdong | 5 | 22°31ʹ31.52ʹʹ | 114°32ʹ27.37ʹʹ |
