# Supplementary material for: Characterization and Development of Microsatellite Markers in Pseudotaxus chienii (Taxaceae) Based on Transcriptome Sequencing
Source: Front Genet. 2020 Oct 15;11:574304. doi: 10.3389/fgene.2020.574304 (PMC7593448; doi:10.3389/fgene.2020.574304)
Supplement: Supplementary Table 3 — The results GO enrichment analysis for unigenes containing microsatellites. [file Table_3.DOCX]

Supplementary Table 3 The result GO enrichment analysis for unigenes containing microsatellite.

| GO ID | GO terms | Annotated | Significant | Expected | P-value | Q-value |
| --- | --- | --- | --- | --- | --- | --- |
| GO:0006351 | transcription, DNA-templated | 3087 | 555 | 345.86 | < 1e-30 | < 1e-30 |
| GO:0032774 | RNA biosynthetic process | 3087 | 555 | 345.86 | < 1e-30 | < 1e-30 |
| GO:0097659 | nucleic acid-templated transcription | 3087 | 555 | 345.86 | < 1e-30 | < 1e-30 |
| GO:0034654 | nucleobase-containing compound biosynthetic process | 3161 | 564 | 354.15 | < 1e-30 | < 1e-30 |
| GO:0006355 | regulation of transcription, DNA-templated | 2526 | 471 | 283.01 | 1.10E-30 | 6.84E-28 |
| GO:1903506 | regulation of nucleic acid-templated transcription | 2526 | 471 | 283.01 | 1.10E-30 | 6.84E-28 |
| GO:2001141 | regulation of RNA biosynthetic process | 2526 | 471 | 283.01 | 1.10E-30 | 6.84E-28 |
| GO:0019438 | aromatic compound biosynthetic process | 3254 | 570 | 364.57 | 7.50E-30 | 3.50E-27 |
| GO:0018130 | heterocycle biosynthetic process | 3363 | 582 | 376.79 | 4.20E-29 | 1.57E-26 |
| GO:0051252 | regulation of RNA metabolic process | 2590 | 474 | 290.18 | 5.90E-29 | 1.68E-26 |
| GO:1901362 | organic cyclic compound biosynthetic process | 3368 | 582 | 377.35 | 6.30E-29 | 1.68E-26 |
| GO:0019219 | regulation of nucleobase-containing compound metabolic process | 2598 | 474 | 291.08 | 1.20E-28 | 2.80E-26 |
| GO:0140110 | transcription regulator activity | 628 | 157 | 65.99 | 2.00E-25 | 2.41E-22 |
| GO:0016070 | RNA metabolic process | 4122 | 664 | 461.82 | 1.60E-24 | 3.32E-22 |
| GO:0006139 | nucleobase-containing compound metabolic process | 5508 | 834 | 617.11 | 8.20E-23 | 1.53E-20 |
| GO:0003700 | DNA-binding transcription factor activity | 573 | 141 | 60.21 | 2.80E-22 | 1.69E-19 |
| GO:0019222 | regulation of metabolic process | 3186 | 529 | 356.95 | 3.60E-22 | 6.11E-20 |
| GO:0060255 | regulation of macromolecule metabolic process | 3136 | 522 | 351.35 | 4.20E-22 | 6.26E-20 |
| GO:0046483 | heterocycle metabolic process | 5698 | 854 | 638.4 | 4.40E-22 | 6.26E-20 |
| GO:0090304 | nucleic acid metabolic process | 5138 | 783 | 575.65 | 4.70E-22 | 6.26E-20 |
| GO:1901360 | organic cyclic compound metabolic process | 5708 | 854 | 639.52 | 7.50E-22 | 9.33E-20 |
| GO:0009987 | cellular process | 16758 | 2151 | 1877.54 | 1.20E-21 | 1.40E-19 |
| GO:0080090 | regulation of primary metabolic process | 3085 | 513 | 345.64 | 1.30E-21 | 1.43E-19 |
| GO:0031323 | regulation of cellular metabolic process | 3110 | 516 | 348.44 | 1.50E-21 | 1.56E-19 |
| GO:0051171 | regulation of nitrogen compound metabolic process | 3080 | 511 | 345.08 | 2.50E-21 | 2.46E-19 |
| GO:0010468 | regulation of gene expression | 2957 | 494 | 331.3 | 3.20E-21 | 2.99E-19 |
| GO:0006725 | cellular aromatic compound metabolic process | 5676 | 843 | 635.93 | 1.30E-20 | 1.16E-18 |
| GO:0009889 | regulation of biosynthetic process | 2904 | 483 | 325.36 | 2.30E-20 | 1.95E-18 |
| GO:0010556 | regulation of macromolecule biosynthetic process | 2901 | 482 | 325.02 | 3.20E-20 | 2.39E-18 |
| GO:0031326 | regulation of cellular biosynthetic process | 2901 | 482 | 325.02 | 3.20E-20 | 2.39E-18 |
| GO:2000112 | regulation of cellular macromolecule biosynthetic process | 2901 | 482 | 325.02 | 3.20E-20 | 2.39E-18 |
| GO:0005667 | transcription factor complex | 1403 | 300 | 181.6 | 1.50E-19 | 8.52E-17 |
| GO:0044271 | cellular nitrogen compound biosynthetic process | 3866 | 606 | 433.14 | 1.80E-19 | 1.29E-17 |
| GO:0065007 | biological regulation | 5226 | 779 | 585.51 | 2.70E-19 | 1.87E-17 |
| GO:0050789 | regulation of biological process | 5038 | 750 | 564.45 | 2.20E-18 | 1.47E-16 |
| GO:0010467 | gene expression | 4467 | 677 | 500.48 | 2.80E-18 | 1.80E-16 |
| GO:0044260 | cellular macromolecule metabolic process | 9488 | 1287 | 1063.02 | 1.40E-17 | 8.71E-16 |
| GO:0034645 | cellular macromolecule biosynthetic process | 4383 | 660 | 491.06 | 3.70E-17 | 2.23E-15 |
| GO:0005488 | binding | 16737 | 2012 | 1758.67 | 4.10E-17 | 1.65E-14 |
| GO:0050794 | regulation of cellular process | 4813 | 714 | 539.24 | 4.50E-17 | 2.62E-15 |
| GO:0009059 | macromolecule biosynthetic process | 4416 | 662 | 494.76 | 9.00E-17 | 5.09E-15 |
| GO:0034641 | cellular nitrogen compound metabolic process | 6182 | 874 | 692.62 | 1.80E-15 | 9.88E-14 |
| GO:1901576 | organic substance biosynthetic process | 5070 | 733 | 568.04 | 6.20E-15 | 3.31E-13 |
| GO:0034599 | cellular response to oxidative stress | 19 | 17 | 2.13 | 9.20E-15 | 4.77E-13 |
| GO:0009058 | biosynthetic process | 5445 | 777 | 610.05 | 1.50E-14 | 7.56E-13 |
| GO:0044249 | cellular biosynthetic process | 5031 | 724 | 563.67 | 2.70E-14 | 1.33E-12 |
| GO:0003779 | actin binding | 259 | 70 | 27.21 | 6.80E-14 | 2.05E-11 |
| GO:0006807 | nitrogen compound metabolic process | 12601 | 1616 | 1411.8 | 2.30E-13 | 1.10E-11 |
| GO:0070887 | cellular response to chemical stimulus | 21 | 17 | 2.35 | 2.60E-13 | 1.21E-11 |
| GO:0043170 | macromolecule metabolic process | 11880 | 1528 | 1331.02 | 8.40E-13 | 3.82E-11 |
| GO:0007010 | cytoskeleton organization | 248 | 67 | 27.79 | 4.30E-12 | 1.91E-10 |
| GO:0044237 | cellular metabolic process | 12838 | 1619 | 1438.35 | 8.60E-11 | 3.73E-09 |
| GO:0008092 | cytoskeletal protein binding | 389 | 84 | 40.87 | 1.10E-10 | 2.65E-08 |
| GO:0006367 | transcription initiation from RNA polymerase II promoter | 90 | 31 | 10.08 | 4.90E-09 | 2.08E-07 |
| GO:0061695 | transferase complex, transferring phosphorus-containing groups | 722 | 147 | 93.45 | 1.10E-08 | 3.12E-06 |
| GO:0006979 | response to oxidative stress | 44 | 19 | 4.93 | 7.20E-08 | 2.99E-06 |
| GO:0042575 | DNA polymerase complex | 145 | 43 | 18.77 | 9.10E-08 | 1.72E-05 |
| GO:0044000 | movement in host | 51 | 20 | 5.71 | 2.30E-07 | 8.10E-06 |
| GO:0046739 | transport of virus in multicellular host | 51 | 20 | 5.71 | 2.30E-07 | 8.10E-06 |
| GO:0046740 | transport of virus in host, cell to cell | 51 | 20 | 5.71 | 2.30E-07 | 8.10E-06 |
| GO:0046794 | transport of virus | 51 | 20 | 5.71 | 2.30E-07 | 8.10E-06 |
| GO:0051814 | movement in other organism involved in symbiotic interaction | 51 | 20 | 5.71 | 2.30E-07 | 8.10E-06 |
| GO:0052126 | movement in host environment | 51 | 20 | 5.71 | 2.30E-07 | 8.10E-06 |
| GO:0052192 | movement in environment of other organism involved in symbiotic interaction | 51 | 20 | 5.71 | 2.30E-07 | 8.10E-06 |
| GO:1902586 | multi-organism intercellular transport | 51 | 20 | 5.71 | 2.30E-07 | 8.10E-06 |
| GO:0003677 | DNA binding | 2078 | 290 | 218.35 | 2.50E-07 | 5.03E-05 |
| GO:0044238 | primary metabolic process | 14442 | 1761 | 1618.06 | 3.30E-07 | 1.14E-05 |
| GO:0003676 | nucleic acid binding | 3839 | 495 | 403.39 | 4.50E-07 | 7.09E-05 |
| GO:0046983 | protein dimerization activity | 289 | 59 | 30.37 | 4.70E-07 | 7.09E-05 |
| GO:0044766 | multi-organism transport | 54 | 20 | 6.05 | 6.70E-07 | 2.23E-05 |
| GO:1902579 | multi-organism localization | 54 | 20 | 6.05 | 6.70E-07 | 2.23E-05 |
| GO:0000428 | DNA-directed RNA polymerase complex | 198 | 51 | 25.63 | 8.40E-07 | 7.95E-05 |
| GO:0016591 | RNA polymerase II, holoenzyme | 198 | 51 | 25.63 | 8.40E-07 | 7.95E-05 |
| GO:0055029 | nuclear DNA-directed RNA polymerase complex | 198 | 51 | 25.63 | 8.40E-07 | 7.95E-05 |
| GO:0005515 | protein binding | 8545 | 1020 | 897.88 | 8.90E-07 | 0.000119 |
| GO:0008686 | 3,4-dihydroxy-2-butanone-4-phosphate synthase activity | 17 | 10 | 1.79 | 1.60E-06 | 0.000193 |
| GO:0016043 | cellular component organization | 1536 | 231 | 172.09 | 1.70E-06 | 5.57E-05 |
| GO:0006996 | organelle organization | 1027 | 161 | 115.06 | 6.50E-06 | 0.000209 |
| GO:0097159 | organic cyclic compound binding | 5250 | 644 | 551.65 | 6.70E-06 | 0.000735 |
| GO:1901363 | heterocyclic compound binding | 5246 | 642 | 551.23 | 9.20E-06 | 0.000925 |
| GO:0071704 | organic substance metabolic process | 14941 | 1796 | 1673.97 | 1.20E-05 | 0.00038 |
| GO:0033926 | glycopeptide alpha-N-acetylgalactosaminidase activity | 24 | 11 | 2.52 | 1.20E-05 | 0.001113 |
| GO:0006811 | ion transport | 926 | 146 | 103.75 | 1.30E-05 | 0.000404 |
| GO:0008914 | leucyltransferase activity | 5 | 5 | 0.53 | 1.30E-05 | 0.00112 |
| GO:0006366 | transcription by RNA polymerase II | 336 | 64 | 37.64 | 1.50E-05 | 0.000459 |
| GO:0004107 | chorismate synthase activity | 29 | 12 | 3.05 | 1.70E-05 | 0.001357 |
| GO:0009664 | plant-type cell wall organization | 5 | 5 | 0.56 | 1.80E-05 | 0.000533 |
| GO:0071669 | plant-type cell wall organization or biogenesis | 5 | 5 | 0.56 | 1.80E-05 | 0.000533 |
| GO:0044798 | nuclear transcription factor complex | 185 | 45 | 23.95 | 1.80E-05 | 0.001278 |
| GO:0090575 | RNA polymerase II transcription factor complex | 185 | 45 | 23.95 | 1.80E-05 | 0.001278 |
| GO:0008270 | zinc ion binding | 777 | 119 | 81.64 | 1.80E-05 | 0.001357 |
| GO:0006468 | protein phosphorylation | 3029 | 407 | 339.36 | 3.70E-05 | 0.001079 |
| GO:0004252 | serine-type endopeptidase activity | 309 | 56 | 32.47 | 3.70E-05 | 0.002625 |
| GO:0000184 | nuclear-transcribed mRNA catabolic process, nonsense-mediated decay | 14 | 8 | 1.57 | 4.00E-05 | 0.001148 |
| GO:0006771 | riboflavin metabolic process | 26 | 11 | 2.91 | 5.30E-05 | 0.001413 |
| GO:0009231 | riboflavin biosynthetic process | 26 | 11 | 2.91 | 5.30E-05 | 0.001413 |
| GO:0016042 | lipid catabolic process | 26 | 11 | 2.91 | 5.30E-05 | 0.001413 |
| GO:0042726 | flavin-containing compound metabolic process | 26 | 11 | 2.91 | 5.30E-05 | 0.001413 |
| GO:0042727 | flavin-containing compound biosynthetic process | 26 | 11 | 2.91 | 5.30E-05 | 0.001413 |
| GO:0050790 | regulation of catalytic activity | 196 | 41 | 21.96 | 5.80E-05 | 0.001524 |
| GO:0046914 | transition metal ion binding | 919 | 134 | 96.57 | 6.10E-05 | 0.004087 |
| GO:0042221 | response to chemical | 207 | 42 | 23.19 | 9.90E-05 | 0.002566 |
| GO:0000079 | regulation of cyclin-dependent protein serine/threonine kinase activity | 62 | 18 | 6.95 | 0.00011 | 0.002701 |
| GO:0071900 | regulation of protein serine/threonine kinase activity | 62 | 18 | 6.95 | 0.00011 | 0.002701 |
| GO:1904029 | regulation of cyclin-dependent protein kinase activity | 62 | 18 | 6.95 | 0.00011 | 0.002701 |
| GO:0009605 | response to external stimulus | 268 | 51 | 30.03 | 0.00011 | 0.002701 |
| GO:0019013 | viral nucleocapsid | 99 | 27 | 12.81 | 0.00011 | 0.006942 |
| GO:0003712 | transcription coregulator activity | 55 | 16 | 5.78 | 0.00012 | 0.007617 |
| GO:0009399 | nitrogen fixation | 16 | 8 | 1.79 | 0.00014 | 0.003393 |
| GO:0065009 | regulation of molecular function | 204 | 41 | 22.86 | 0.00015 | 0.003588 |
| GO:0016838 | carbon-oxygen lyase activity, acting on phosphates | 35 | 12 | 3.68 | 0.00015 | 0.009045 |
| GO:0060962 | regulation of ribosomal protein gene transcription by RNA polymerase II | 4 | 4 | 0.45 | 0.00016 | 0.003779 |
| GO:0004659 | prenyltransferase activity | 57 | 16 | 5.99 | 0.00018 | 0.010337 |
| GO:0140103 | catalytic activity, acting on a glycoprotein | 36 | 12 | 3.78 | 0.0002 | 0.010964 |
| GO:0016310 | phosphorylation | 3159 | 415 | 353.93 | 0.00021 | 0.004898 |
| GO:1990234 | transferase complex | 1434 | 231 | 185.61 | 0.00021 | 0.011928 |
| GO:0019220 | regulation of phosphate metabolic process | 100 | 24 | 11.2 | 0.00023 | 0.005234 |
| GO:0051174 | regulation of phosphorus metabolic process | 100 | 24 | 11.2 | 0.00023 | 0.005234 |
| GO:0046872 | metal ion binding | 1993 | 258 | 209.42 | 0.00023 | 0.01206 |
| GO:0071840 | cellular component organization or biogenesis | 1941 | 266 | 217.47 | 0.00026 | 0.005845 |
| GO:0005672 | transcription factor TFIIA complex | 68 | 20 | 8.8 | 0.00027 | 0.013942 |
| GO:0006189 | 'de novo' IMP biosynthetic process | 10 | 6 | 1.12 | 0.00028 | 0.006147 |
| GO:0019915 | lipid storage | 10 | 6 | 1.12 | 0.00028 | 0.006147 |
| GO:0001932 | regulation of protein phosphorylation | 96 | 23 | 10.76 | 0.00031 | 0.006427 |
| GO:0042325 | regulation of phosphorylation | 96 | 23 | 10.76 | 0.00031 | 0.006427 |
| GO:0043549 | regulation of kinase activity | 96 | 23 | 10.76 | 0.00031 | 0.006427 |
| GO:0045859 | regulation of protein kinase activity | 96 | 23 | 10.76 | 0.00031 | 0.006427 |
| GO:0051338 | regulation of transferase activity | 96 | 23 | 10.76 | 0.00031 | 0.006427 |
| GO:0043169 | cation binding | 2006 | 258 | 210.78 | 0.00034 | 0.017085 |
| GO:0051701 | interaction with host | 192 | 38 | 21.51 | 0.00035 | 0.007099 |
| GO:0006401 | RNA catabolic process | 22 | 9 | 2.46 | 0.00035 | 0.007099 |
| GO:0000221 | vacuolar proton-transporting V-type ATPase, V1 domain | 45 | 15 | 5.82 | 0.00035 | 0.014555 |
| GO:0016471 | vacuolar proton-transporting V-type ATPase complex | 45 | 15 | 5.82 | 0.00035 | 0.014555 |
| GO:0017004 | cytochrome complex assembly | 41 | 13 | 4.59 | 0.00036 | 0.007223 |
| GO:0004014 | adenosylmethionine decarboxylase activity | 28 | 10 | 2.94 | 0.00036 | 0.017366 |
| GO:0001503 | ossification | 14 | 7 | 1.57 | 0.00037 | 0.007268 |
| GO:0065003 | protein-containing complex assembly | 352 | 61 | 39.44 | 0.00037 | 0.007268 |
| GO:0042151 | nematocyst | 16 | 8 | 2.07 | 0.00038 | 0.014555 |
| GO:0071203 | WASH complex | 32 | 12 | 4.14 | 0.0004 | 0.014555 |
| GO:0043231 | intracellular membrane-bounded organelle | 5806 | 829 | 751.52 | 0.00041 | 0.014555 |
| GO:0003911 | DNA ligase (NAD+) activity | 15 | 7 | 1.58 | 0.00042 | 0.019482 |
| GO:0040011 | locomotion | 148 | 31 | 16.58 | 0.00043 | 0.008358 |
| GO:0050896 | response to stimulus | 2685 | 354 | 300.82 | 0.00049 | 0.009424 |
| GO:0006796 | phosphate-containing compound metabolic process | 3628 | 467 | 406.48 | 0.0005 | 0.009424 |
| GO:0031399 | regulation of protein modification process | 99 | 23 | 11.09 | 0.0005 | 0.009424 |
| GO:0043933 | protein-containing complex subunit organization | 358 | 61 | 40.11 | 0.00058 | 0.010663 |
| GO:0000956 | nuclear-transcribed mRNA catabolic process | 19 | 8 | 2.13 | 0.00059 | 0.010663 |
| GO:0006402 | mRNA catabolic process | 19 | 8 | 2.13 | 0.00059 | 0.010663 |
| GO:0005774 | vacuolar membrane | 47 | 15 | 6.08 | 0.00059 | 0.018618 |
| GO:0044437 | vacuolar part | 47 | 15 | 6.08 | 0.00059 | 0.018618 |
| GO:0006793 | phosphorus metabolic process | 3636 | 467 | 407.37 | 0.0006 | 0.010663 |
| GO:0030682 | evasion or tolerance of host defense response | 28 | 10 | 3.14 | 0.0006 | 0.010663 |
| GO:0051807 | evasion or tolerance of defense response of other organism involved in symbiotic interaction | 28 | 10 | 3.14 | 0.0006 | 0.010663 |
| GO:0005669 | transcription factor TFIID complex | 88 | 23 | 11.39 | 0.00064 | 0.019133 |
| GO:0043207 | response to external biotic stimulus | 205 | 39 | 22.97 | 0.00066 | 0.01151 |
| GO:0051707 | response to other organism | 205 | 39 | 22.97 | 0.00066 | 0.01151 |
| GO:0016830 | carbon-carbon lyase activity | 270 | 46 | 28.37 | 0.0007 | 0.031267 |
| GO:0016071 | mRNA metabolic process | 247 | 45 | 27.67 | 0.00071 | 0.012267 |
| GO:0032501 | multicellular organismal process | 399 | 66 | 44.7 | 0.00081 | 0.013191 |
| GO:0044413 | avoidance of host defenses | 29 | 10 | 3.25 | 0.00082 | 0.013191 |
| GO:0044415 | evasion or tolerance of host defenses | 29 | 10 | 3.25 | 0.00082 | 0.013191 |
| GO:0051832 | avoidance of defenses of other organism involved in symbiotic interaction | 29 | 10 | 3.25 | 0.00082 | 0.013191 |
| GO:0051834 | evasion or tolerance of defenses of other organism involved in symbiotic interaction | 29 | 10 | 3.25 | 0.00082 | 0.013191 |
| GO:0052173 | response to defenses of other organism | 29 | 10 | 3.25 | 0.00082 | 0.013191 |
| GO:0052200 | response to host defenses | 29 | 10 | 3.25 | 0.00082 | 0.013191 |
| GO:0075136 | response to host | 29 | 10 | 3.25 | 0.00082 | 0.013191 |
| GO:0070461 | SAGA-type complex | 14 | 7 | 1.81 | 0.0009 | 0.02556 |
| GO:0006464 | cellular protein modification process | 4381 | 552 | 490.84 | 0.00105 | 0.016604 |
| GO:0036211 | protein modification process | 4381 | 552 | 490.84 | 0.00105 | 0.016604 |
| GO:0003909 | DNA ligase activity | 17 | 7 | 1.79 | 0.00105 | 0.045225 |
| GO:0005201 | extracellular matrix structural constituent | 13 | 6 | 1.37 | 0.0012 | 0.049848 |
| GO:0004512 | inositol-3-phosphate synthase activity | 27 | 9 | 2.84 | 0.00124 | 0.049848 |
| GO:0071941 | nitrogen cycle metabolic process | 21 | 8 | 2.35 | 0.0013 | 0.020385 |
| GO:0034622 | cellular protein-containing complex assembly | 276 | 48 | 30.92 | 0.00136 | 0.021148 |
| GO:1902494 | catalytic complex | 2147 | 324 | 277.9 | 0.00137 | 0.037055 |
| GO:0005773 | vacuole | 51 | 15 | 6.6 | 0.00152 | 0.039244 |
| GO:0022607 | cellular component assembly | 492 | 77 | 55.12 | 0.0016 | 0.024674 |
| GO:0005739 | mitochondrion | 901 | 147 | 116.62 | 0.0017 | 0.041983 |
| GO:0033554 | cellular response to stress | 330 | 55 | 36.97 | 0.00176 | 0.026919 |
| GO:0044423 | virion part | 792 | 131 | 102.52 | 0.00178 | 0.042127 |
| GO:0017053 | transcriptional repressor complex | 28 | 10 | 3.62 | 0.00186 | 0.042245 |
| GO:0045595 | regulation of cell differentiation | 6 | 4 | 0.67 | 0.00196 | 0.029735 |
| GO:0005834 | heterotrimeric G-protein complex | 24 | 9 | 3.11 | 0.00211 | 0.042245 |
| GO:0031234 | extrinsic component of cytoplasmic side of plasma membrane | 24 | 9 | 3.11 | 0.00211 | 0.042245 |
| GO:1905360 | GTPase complex | 24 | 9 | 3.11 | 0.00211 | 0.042245 |
| GO:0043227 | membrane-bounded organelle | 5974 | 840 | 773.27 | 0.00216 | 0.042245 |
| GO:0002115 | store-operated calcium entry | 18 | 7 | 2.02 | 0.00228 | 0.033238 |
| GO:0010959 | regulation of metal ion transport | 18 | 7 | 2.02 | 0.00228 | 0.033238 |
| GO:0043269 | regulation of ion transport | 18 | 7 | 2.02 | 0.00228 | 0.033238 |
| GO:0051924 | regulation of calcium ion transport | 18 | 7 | 2.02 | 0.00228 | 0.033238 |
| GO:2001256 | regulation of store-operated calcium entry | 18 | 7 | 2.02 | 0.00228 | 0.033238 |
| GO:0005942 | phosphatidylinositol 3-kinase complex | 16 | 7 | 2.07 | 0.00238 | 0.042245 |
| GO:0005944 | phosphatidylinositol 3-kinase complex, class IB | 16 | 7 | 2.07 | 0.00238 | 0.042245 |
| GO:0097651 | phosphatidylinositol 3-kinase complex, class I | 16 | 7 | 2.07 | 0.00238 | 0.042245 |
| GO:1905348 | endonuclease complex | 91 | 22 | 11.78 | 0.00248 | 0.042686 |
| GO:0006890 | retrograde vesicle-mediated transport, Golgi to endoplasmic reticulum | 33 | 10 | 3.7 | 0.0025 | 0.036163 |
| GO:0070070 | proton-transporting V-type ATPase complex assembly | 28 | 9 | 3.14 | 0.0026 | 0.036767 |
| GO:0070072 | vacuolar proton-transporting V-type ATPase complex assembly | 28 | 9 | 3.14 | 0.0026 | 0.036767 |
| GO:0006188 | IMP biosynthetic process | 14 | 6 | 1.57 | 0.00266 | 0.036767 |
| GO:0007606 | sensory perception of chemical stimulus | 14 | 6 | 1.57 | 0.00266 | 0.036767 |
| GO:0046040 | IMP metabolic process | 14 | 6 | 1.57 | 0.00266 | 0.036767 |
| GO:0050909 | sensory perception of taste | 14 | 6 | 1.57 | 0.00266 | 0.036767 |
| GO:0006270 | DNA replication initiation | 80 | 18 | 8.96 | 0.00284 | 0.038966 |
| GO:0006950 | response to stress | 1021 | 143 | 114.39 | 0.00293 | 0.039908 |
| GO:0009898 | cytoplasmic side of plasma membrane | 25 | 9 | 3.24 | 0.00293 | 0.046229 |
| GO:0098552 | side of membrane | 25 | 9 | 3.24 | 0.00293 | 0.046229 |
| GO:0098562 | cytoplasmic side of membrane | 25 | 9 | 3.24 | 0.00293 | 0.046229 |
| GO:0043412 | macromolecule modification | 4462 | 555 | 499.92 | 0.00296 | 0.040024 |
| GO:0006813 | potassium ion transport | 81 | 18 | 9.08 | 0.00328 | 0.044032 |
| GO:0051336 | regulation of hydrolase activity | 75 | 17 | 8.4 | 0.00338 | 0.045051 |
| GO:0006261 | DNA-dependent DNA replication | 82 | 18 | 9.19 | 0.00377 | 0.049892 |
