# Supplementary material for: Characterization and Development of Microsatellite Markers in Pseudotaxus chienii (Taxaceae) Based on Transcriptome Sequencing
Source: Front Genet. 2020 Oct 15;11:574304. doi: 10.3389/fgene.2020.574304 (PMC7593448; doi:10.3389/fgene.2020.574304)
Supplement: Supplementary Table 4 — The 77 primers pairs of P. chienii screened in this study. [file Table_4.DOCX]

Supplementary Table 4 The 77 primers pairs of *P. chienii* screened in this study.

| Locus | Primer sequence (5’-3’) | Repeat motif | Size range (bp) | Tm (℃) | Polymorphism level | GenBank accession no. |
| --- | --- | --- | --- | --- | --- | --- |
| *P. chienii*_10 | F: CATTAGGACAGGAGCAGGAC  R: TTGCGGGACAGTAGACAGA | (AAACTA)6 | 432 | 54 | polymorphism | MT563345 |
| *P. chienii*_11 | F: GCTTTGGAATGACGCTGAT  R: ATTTGTTGGGAGGTGGGTA | (AAGTCT)5 | 472 | 55 | monomorphism | MT563346 |
| *P. chienii*_17 | F: GGTCGAGTACGTGGTGGTTT  R: GCCTGCGCTGTCATAAACTG | (AGG)5 | 161 | 57 | polymorphism | MT563347 |
| *P. chienii*_18 | F: TATTGGATCCGACGTGCTGG  R: TCTCTTGATGGCTGCGAAGG | (TC)6 | 142 | 60 | monomorphism | MT563348 |
| *P. chienii*_19 | F: ACATGGTTGGCATCAGCATC  R: GCTCCCTCGTTCCCTTCAAT | (TC)12 | 105 | 59 | polymorphism | MT563349 |
| *P. chienii*_20 | F: TGTGCCAGTACTGCTACTGC  R: TGAATGCGTGCGGAAACAAG | (ACC)6 | 185 | 57 | polymorphism | MT563350 |
| *P. chienii*_21 | F: TGTGCCAGTACTGCTACTGC  R: GCGGAAACAAGGCAATCCTC | (ACC)6 | 176 | 59 | polymorphism | MT563345 |
| *P. chienii*_25 | F: GATGCCGCTGGTTTCAATCC  R: GCCGTACCGATTGGGATCAT | (GGA)8 | 207 | 57 | polymorphism | MT563351 |
| *P. chienii*_28 | F: GAGTGGGAGACGAAGAGTGC  R: CGAAGTGGGCTGCAACAATG | (CTC)8 | 260 | 57 | polymorphism | MT563352 |
| *P. chienii*_29 | F: AGCTGCAAGGCTACACAGAG  R: CAATCCCGGGCCTGTTAGAA | (GAA)5 | 239 | 57 | monomorphism | MT563353 |
| *P. chienii*_30 | F: CCGAGCATACGCGTCTTTTG  R: CATGCCCTGCCACAATCAAG | (CCT)7 | 208 | 59 | monomorphism | MT563354 |
| *P. chienii*_31 | F: GGCCTCTGCAGTCATGTTCT  R: GGTGAAAGGCCGTTTGCAAT | (CCT)6 | 107 | 59 | polymorphism | MT563355 |
| *P. chienii*_32 | F: CGAAGGCCCTCTTGAGTTGT  R: GGAAACCTGCCATACGTTGC | (ATC)5 | 194 | 59 | monomorphism | MT563356 |
| *P. chienii*_34 | F: GGCTCGCTGCAAAACATGAA  R: TCTTCCACTCCTGGGACCAT | (AGA)6 | 249 | 60 | monomorphism | MT563357 |
| *P. chienii*_36 | F: GGGCCATCCTCTTCCTCAAC  R: CTCGACACTGCTCCACATCT | (TCC)8 | 244 | 57 | polymorphism | MT563358 |
| *P. chienii*_38 | F: ACCGCTTCAAGTAGTACGGC  R: GCCAGAAGGTACCAGGCATT | (TTA)8 | 237 | 60 | polymorphism | MT563359 |
| *P. chienii*_39 | F: CTCAGATCACCCTAACCCGC  R: ACCCCGGAAAATAGTCCTGC | (TCT)5 | 226 | 57 | monomorphism | MT563360 |
| *P. chienii*_40 | F: GGCCTCCTCATCACAGCTAC  R: CCAGCTTCTCCTCGTTCGAG | (TCT)8 | 280 | 59 | polymorphism | MT563361 |
| *P. chienii*_41 | F: TGAAATCGGCCCAGAACCTG  R: TTGCTGGAAGCCAATCCCAT | (GAT)5 | 190 | 59 | monomorphism | MT563362 |
| *P. chienii*_45 | F: TCTTCCTTGCGAAGTGGAGC  R: GGTGGGCGGTATGATCGATT | (AAT)5 | 242 | 59 | polymorphism | MT563363 |
| *P. chienii*_51 | F: TGTGCCAGTACTGCTACTGC  R: GCTTCTCTGCTGCTCCAGAA | (CATTCA)5 | 263 | 57 | polymorphism | MT563350 |
| *P. chienii*_52 | F: TTTGTTCTCCTTGCCCCAGG  R: GCTTCTCTGCTGCTCCAGAA | (CATTCA)6 | 211 | 59 | polymorphism | MT563345 |
| *P. chienii*_53 | F: AGCACTTTCGACAGAGCCTC  R: TTCCCTTCCCACACCCAAAC | (CAGGTT)6 | 219 | 60 | polymorphism | MT563364 |
| *P. chienii*_54 | F: TCCTCGCAGGACAATGCATT  R: GCAATTCATGGACACGACCG | (GGCATC)5 | 219 | 57 | polymorphism | MT563365 |
| *P. chienii*_56 | F: AATTGGTCACTCACCCGCAT  R: CTCATATGGTAATTAGCAGCCTGC | (AAATCC)5 | 191 | 59 | monomorphism | MT563366 |
| *P. chienii*_63 | F: TGTGGATGATGGTGGAGTT  R: TCGCACGACGATTCTGATA | (AGG)8 | 218 | 55 | polymorphism | MT563367 |
| *P. chienii*_74 | F: CGCTCCAACGAATCCAACC  R: ATGCCATCCGCACAACCTC | (CAGAAG)5 | 265 | 57 | polymorphism | MT563368 |
| *P. chienii*_75 | F: CGCTCCAACGAATCCAACC  R: TAATGCCATCCGCACAACC | (CAGAAG)5 | 267 | 57 | polymorphism | MT563368 |
| *P. chienii*_84 | F: AGTCGCTCCAACGAATCCAA  R: TTGAGTAGCAACGGACCCAC | (CAGAAG)5 | 109 | 57 | polymorphism | MT563368 |
| *P. chienii*_86 | F: GAATTTGAAGCACGGCCTCA  R: GAGTGCCCTGCTTTCTGGAT | (GGCACC)5 | 278 | 57 | polymorphism | MT563369 |
| *P. chienii*_97 | F: TAAGGAAAGCAGCTCGCCAA  R: GGTTGGAGTGGAGGTGACTG | (GGA)5 | 160 | 59 | monomorphism | MT563370 |
| *P. chienii*_103 | F: AAGTGCTGTCGAGAATGGCA  R: TCGTGTTCGACCATCGCTAG | (GGC)6 | 181 | 55 | monomorphism | MT563370 |
| *P. chienii*_115 | F: GTTGGCGCAATCCAGTTGTT  R: AGTGACGTTGGCCTGTGAAA | (TCC)5 | 222 | 56 | monomorphism | MT563370 |
| *P. chienii*_118 | F: GGATGATGGTGGAGTTTTTCGT  R: ACTTGCCTGGGCTTAAGACC | (AGG)8 | 186 | 57 | polymorphism | MT563367 |
| *P. chienii*_123 | F: CCGAGATGTGATGTGGTCGT  R: GCCTATTGGGTCTCACATGC | (CTC)5 | 251 | 57 | monomorphism | MT563371 |
| *P. chienii*_124 | F: CCCACTCGTCCTGCTTTCTT  R: GAGATGACAAGCGCAGAAGC | (CTC)5 | 173 | 57 | monomorphism | MT563372 |
| *P. chienii*_131 | F: AGCCATAAAACCTGCCTGCT  R: CTCCTTCCAGGGGAGCAAAG | (CAC)7(CTC)6 | 167 | 57 | monomorphism | MT563373 |
| *P. chienii*_134 | F: ACGCCACGTTAGGACACAAT  R: CCTAGATCAAGAGCGGCCTG | (CTT)6 | 273 | 57 | polymorphism | MT563374 |
| *P. chienii*_136 | F: CTAGCAGAGGTGGTGGCATC  R: GACACCCATCCCTGAGAAGC | (TCT)5 | 246 | 55 | monomorphism | MT563375 |
| *P. chienii*_138 | F: CTGCTCCAGTGGATGCTGAG  R: GCATCCCTGTGTTCCTCGAT | (TGA)8 | 157 | 56 | monomorphism | MT563376 |
| *P. chienii*_141 | F: CTGTCAACAAGCGGCTTTCC  R: AGAGCCGGGGGAAAATTGAG | (CGG)7 | 238 | 57 | polymorphism | MT563377 |
| *P. chienii*_152 | F: CCCATCTGAACCCACGCTAA  R: AAAGCGCTCATGCCCAAAAC | (GGC)7 | 246 | 57 | polymorphism | MT563378 |
| *P. chienii*_162 | F: ACCTATCACCTCCTCGACCC  R: CCGTTCCATCACTGTGGACA | (CCACCG)6 | 230 | 55 | polymorphism | MT563379 |
| *P. chienii*_163 | F: ACCTATCACCTCCTCGACCC  R: TCGTGGTTATGGTGGTGGTG | (CCACCG)6 | 164 | 57 | polymorphism | MT563379 |
| *P. chienii*_192 | F: GTCCTCCTCCCTCCTCAACA  R: ACACGCTTTCCATGTCGACT | (CGC)5 | 257 | 57 | monomorphism | MT563380 |
| *P. chienii*_193 | F: TGTCCTCCTCCCTCCTCAAC  R: ACACGCTTTCCATGTCGACT | (CGC)5 | 258 | 56 | monomorphism | MT563380 |
| *P. chienii*_194 | F: GTCCTCCTCCCTCCTCAACA  R: CGCAGTTGGAAGAGGGTTCT | (CGC)5 | 150 | 57 | monomorphism | MT563380 |
| *P. chienii*_198 | F: GAGGGATACAGAAGCACAG  R: TATGACAAACCCAAACGAG | (ATA)5 | 287 | 56 | polymorphism | MT563329 |
| *P. chienii*_200 | F: CACCACATGCTGCTCTGCTGAAC  R: GAATCCACGCCTTTGAACTCGC | (ATC)6(AAG)6 | 286 | 58 | monomorphism | MT563330 |
| *P. chienii*_203 | F: TAACCAGAGGGAGTGAGT  R: CAATAAGAAGTCGGTACAGTG | (AAC)6 | 269 | 56 | monomorphism | MT563331 |
| *P. chienii*_204 | F: TGGTGGAAGTGTGCCAAATG  R: GGAGCAGCAGAAGAAAGAAC | (ATGG)6 | 127 | 58 | monomorphism | MT563332 |
| *P. chienii*_209 | F: CCGTCACAGGTTCACAGATG  R: TATTGCGAGAAAGAGTAGATG | (AT)7(GT)8 | 335 | 58 | monomorphism | MT563333 |
| *P. chienii*_214 | F: GACAACGGCAAAGGAGGAAT  R: GCGATAGCCACCAAAGACAT | (ATA)6 | 321 | 58 | polymorphism | MT563334 |
| *P. chienii*_216 | F: TGCGGTTCAGTAACAGTCCTTC  R: TCCCCCACCTCTTCCCAG | (CTCCTG)5 | 440 | 58 | polymorphism | MT563335 |
| *P. chienii*_227 | F: TGATACTTGTATGCCCTCTG  R: ATTCCTCATCTATCTCCCAC | (AAG)6 | 401 | 56 | monomorphism | MT563336 |
| *P. chienii*_233 | F: TGTGTGAAAGGACAAGGCGT  R: GCACCCTATTCACCCGAGAT | (ATGCAG)7 | 249 | 56 | polymorphism | MT563337 |
| *P. chienii*_234 | F: ACAATCCAAGGAGCTTAAGT  R: CCAGGTTCGCAATACAAT | (CA)9 | 370 | 54 | monomorphism | MT563338 |
| *P. chienii*_242 | F: GTAAACAAAGGAGGACGGTG  R: TACGGAGCACCCGACAGT | (GAT)5 | 351 | 58 | polymorphism | MT563339 |
| *P. chienii*_243 | F: TTGTGGGTTTTCCTTTGACG  R: CACGCAAGTACAACACGGTC | (ATA)5 | 156 | 58 | monomorphism | MT563340 |
| *P. chienii*_247 | F: CACATGGTGCGTGTTATTAG  R: AGGCTATTGCTGGTTTGG | (A)10ca(T)10 | 413 | 58 | polymorphism | MT563341 |
| *P. chienii*_249 | F: CATGCCCGCACACTTCAAAT  R: TTCCTCTCTCTGTCCCCACC | (AT)7 | 259 | 60 | polymorphism | MT563342 |
| *P. chienii*_251 | F: CAAAAGCGCTGACGTGGATG  R: GATCTGCTGTCCCATAGGCT | (AAG)6 | 141 | 60 | monomorphism | MT563343 |
| *P. chienii*_259 | F: GTCCCTCCAGCGTACTATGC  R: CCTTGAGTGCCGGTAATTGC | (ATA)6 | 217 | 60 | polymorphism | MT563334 |
| *P. chienii*_263 | F: CCCTCTGAGTGGCCATGTTT  R: TCACCAACCCTGCATTGTGA | (AAG)6 | 244 | 60 | monomorphism | MT563336 |
| *P. chienii*_265 | F: CTCTCCCTCTCACTCCCGTT  R: CGCGTCGATCAGCATCAAAG | (GCG)5 | 174 | 60 | monomorphism | MT563344 |
| *P. chienii*_267 | F: CCCCTCATTGACAGGTTC  R: AAGATAGTCGGGACACCAAG | (CTG)5 | 311 | 56 | polymorphism | MT563381 |
| *P. chienii*_270 | F: TCCTCACAAGCGTAATAAGAC  R: TCAAATACAGCCAACCTCAT | (CTC)5 | 425 | 52 | monomorphism | MT563382 |
| *P. chienii*_272 | F: TGGAGGGTTGTTTATCGT  R: AATGGTGGAAGTGAATGAG | (ATC)7 | 376 | 54 | monomorphism | MT563383 |
| *P. chienii*_276 | F: TTTCATTGTGTCTACCCTTGTG  R: GCATTATTTTGGAGATTCGT | (ATC)5 | 273 | 54 | polymorphism | MT563384 |
| *P. chienii*_281 | F: CGGCTTCGGCTTCTGCTT  R: AGACGGGCGTGGATTGCT | (TTA)5 | 415 | 60 | monomorphism | MT563385 |
| *P. chienii*_288 | F: CACGCCCACCATAGTTGT  R: GGAGGAAGATGTCGTTGAAG | (AAGG)5 | 261 | 58 | polymorphism | MT563386 |
| *P. chienii*_305 | F: CTGCGTCACTGATGGCTTTG  R: CCAAACAGGAGGAACGGACA | (GCG)7 | 115 | 60 | monomorphism | MT563387 |
| *P. chienii*_306 | F: GGCCTCATGTTCATGCCTCT  R: GGGCAGGAATTTGCTACCCT | (GAA)6 | 110 | 60 | monomorphism | MT563388 |
| *P. chienii*_317 | F: TGAACAACGATGGGCGAAGA  R: CGAAGTGAGCCTGTTTGCAC | (AG)7 | 183 | 60 | monomorphism | MT563389 |
| *P. chienii*_341 | F: GACCTCTTACCAGCTGCGAG  R: ACCACCGGTTTCAGTTTCGT | (CCT)11 | 220 | 62 | polymorphism | MT563390 |
| *P. chienii*_354 | F: CCTTGTGTTTGTGGTGCTCG  R: AGGGTGACTTGCAAGGCTAC | (ATC)5 | 207 | 62 | monomorphism | MT563391 |
| *P. chienii*_358 | F: TAAGTGGCTGCTGCATCACA  R: TACAGCAGCAGCAGAGCTTT | (TCC)5 | 249 | 58 | polymorphism | MT563392 |
