# Supplementary material for: Characterization and Development of Microsatellite Markers in Pseudotaxus chienii (Taxaceae) Based on Transcriptome Sequencing
Source: Front Genet. 2020 Oct 15;11:574304. doi: 10.3389/fgene.2020.574304 (PMC7593448; doi:10.3389/fgene.2020.574304)
Supplement: Supplementary Table 5 — Bottleneck analysis for four populations of P. chienii. [file Table_5.DOCX]

Supplementary Table 5 Bottleneck analysis for four populations of *P. chienii.*

|  | Sign test | | | | | | Wilcoxon sign rank test | | |
| --- | --- | --- | --- | --- | --- | --- | --- | --- | --- |
| Pop | IAM | | TPM | | SMM | | IAM | TPM | SMM |
|  | H_E_/H_D_ | P | H_E_/H_D_ | P | H_E_/H_D_ | P | P | P | P |
| MS | 10/8 | 0.471 | 8/10 | 0.263 | 7/11 | 0.099 | 0.417 | 0.899 | 0.090 |
| YS | 10/7 | 0.396 | 8/9 | 0.325 | 5/12 | 0.021^*^ | 0.678 | 0.284 | 0.015^*^ |
| BJS | 13/7 | 0.144 | 8/12 | 0.169 | 5/15 | 0.007^**^ | 0.546 | 0.261 | 0.009^**^ |
| ZJJ | 10/7 | 0.365 | 9/8 | 0.571 | 8/9 | 0.318 | 0.431 | 0.579 | 0.159 |

Note: IAM: Infinite Allele Model; TPM: Two-phased Mutation Model; SMM: Stepwise Mutation Model. H_E_/H_D_: Ratio of loci number with heterozygosity excess to heterozygosity deficiency, P: The significance level by sign test and wilcoxon sign rank test. * 0.05<P<0.01, ** P<0.01.
