# Supplementary material for: Characterization and Development of Microsatellite Markers in Pseudotaxus chienii (Taxaceae) Based on Transcriptome Sequencing
Source: Front Genet. 2020 Oct 15;11:574304. doi: 10.3389/fgene.2020.574304 (PMC7593448; doi:10.3389/fgene.2020.574304)
Supplement: Supplementary Table 6 — PCR amplification results of 12 pairs of polymorphic SSR primers in Amentotaxus argotaenia. [file Table_6.DOCX]

Supplementary Table 6 PCR amplification results of 12 pairs of polymorphic SSR primers in *Amentotaxus argotaenia.*

| Locus | Na | Ne | I | Ho | He | PIC | Size range  (bp) |
| --- | --- | --- | --- | --- | --- | --- | --- |
| *P. chienii*-20 | 7.000 | 2.492 | 1.136 | 0.828 | 0.599 | 0.528 | 183-199 |
| *P. chienii*-25 | 2.000 | 1.665 | 0.589 | 0.000 | 0.400 | 0.320 | 198-199 |
| *P. chienii*-29 | 3.000 | 1.279 | 0.441 | 0.000 | 0.218 | 0.205 | 169-239 |
| *P. chienii*-36 | 2.000 | 1.187 | 0.294 | 0.000 | 0.158 | 0.145 | 246-247 |
| *P. chienii*-75 | 7.000 | 3.867 | 1.539 | 0.983 | 0.741 | 0.701 | 253-266 |
| *P. chienii*-134 | 3.000 | 1.405 | 0.494 | 0.034 | 0.288 | 0.251 | 273-275 |
| *P. chienii*-141 | 5.000 | 1.726 | 0.750 | 0.500 | 0.421 | 0.363 | 232-242 |
| *P. chienii*-152 | 7.000 | 3.611 | 1.468 | 1.000 | 0.723 | 0.684 | 242-290 |
| *P. chienii*-162 | 5.000 | 2.054 | 1.029 | 0.276 | 0.513 | 0.481 | 209-216 |
| *P. chienii*-198 | 3.000 | 1.338 | 0.449 | 0.017 | 0.253 | 0.225 | 280-288 |
| *P. chienii*-341 | 4.000 | 2.617 | 1.107 | 0.793 | 0.618 | 0.558 | 208-218 |
| *P. chienii*-358 | 2.000 | 1.153 | 0.257 | 0.036 | 0.133 | 0.124 | 250-251 |
| Mean | 4.167 | 2.033 | 0.796 | 0.372 | 0.422 | 0.382 | - |

Note: Na: Number of alleles; Ne: Number of effective alleles; Ho: Observed heterozygosity; He: Expected heterozygosity; I: Shannon’s information index; PIC: Polymorphism information content.
